# Post-Disturbance Stability of Fish Assemblages Measured at Coarse Taxonomic Resolution Masks Change at Finer Scales

**DOI:** 10.1371/journal.pone.0156232

**Published:** 2016-06-10

**Authors:** Daniela M. Ceccarelli, Michael J. Emslie, Zoe T. Richards

**Affiliations:** 1 ARC Centre of Excellence for Coral Reef Studies, James Cook University, Douglas, QLD, 4811, Townsville, Australia; 2 Australian Institute of Marine Science, PMB No. 3, TMC, Townsville, QLD, 4810, Australia; 3 Department of Aquatic Zoology, Western Australian Museum, 49 Kew Street, Welshpool, WA, 6016, Perth, Australia; Department of Agriculture and Water Resources, AUSTRALIA

## Abstract

Quantifying changes to coral reef fish assemblages in the wake of cyclonic disturbances is challenging due to spatial variability of damage inherent in such events. Often, fish abundance appears stable at one spatial scale (e.g. reef-wide), but exhibits substantial change at finer scales (e.g. site-specific decline or increase). Taxonomic resolution also plays a role; overall stability at coarse taxonomic levels (e.g. family) may mask species-level turnover. Here we document changes to reef fish communities after severe Tropical Cyclone Ita crossed Lizard Island, Great Barrier Reef. Coral and reef fish surveys were conducted concurrently before and after the cyclone at four levels of exposure to the prevailing weather. Coral cover declined across all exposures except sheltered sites, with the largest decline at exposed sites. There was no significant overall reduction in the total density, biomass and species richness of reef fishes between 2011 and 2015, but individual fish taxa (families and species) changed in complex and unpredictable ways. For example, more families increased in density and biomass than decreased following Cyclone Ita, particularly at exposed sites whilst more fish families declined at lagoon sites even though coral cover did not decline. All sites lost biomass of several damselfish species, and at most sites there was an increase in macroinvertivores and grazers. Overall, these results suggest that the degree of change measured at coarse taxonomic levels masked high species-level turnover, although other potential explanations include that there was no impact of the storm, fish assemblages were impacted but underwent rapid recovery or that there is a time lag before the full impacts become apparent. This study confirms that in high-complexity, high diversity ecosystems such as coral reefs, species level analyses are essential to adequately capture the consequences of disturbance events.

## Introduction

Cyclones are large meteorological events that can have variable effects on coral reef communities, from minimal impacts [1, 2] to severe damage [3, 4]. While storm swell associated with these events can reach upwards of 10m (33ft), damage to benthic communities can vary around individual reefs, due to shifts in wind direction (and associated storm swell) as a cyclone passes any given point [5, 6]. This means that cyclone damage will affect different parts of a reef at different times and importantly, sites normally sheltered from the prevailing weather may be heavily impacted by cyclones. Effects of tropical cyclones and storms include extensive physical damage to coral communities and the underlying reef matrix, resuspension of sediments and increased turbidity leading to coral mortality and sub-lethal effects through smothering and reduced light levels [7, 8]. In some cases, rotting organic matter can lead to pulses of nutrient enrichment in lagoonal environments [5]. Additionally, heavy rainfall associated with these systems can cause freshwater inundation of coral reefs and associated bleaching and mortality of coral colonies. Different benthic taxa have varying susceptibility to cyclones, depending on their morphology and their location on the reef, with fragile branching and plate-like forms more susceptible to damage than more robust massive growth forms [9, 10]. More delicate coral morphologies are generally found in sheltered environments, such as lagoons or the leeward sides of reefs, which are rarely impacted by the prevailing weather conditions. Due to their nature, cyclones have the potential to impact such sites as the wind and associated swells change direction as a cyclone passes. However, it is rare for a study to document the effects of a severe tropical cyclone on reef communities across different degrees of wave exposure.

Just as cyclonic effects to the benthos can vary, recent research has highlighted that coral reef fish communities also have variable responses to various forms of habitat degradation, including cyclones [11, 12, 13, 14]. The general consensus is that if the three-dimensional structural complexity of the reef framework is maintained, the fish assemblage should stay relatively stable [15, 16, 17, 18]. A variety of finer-scale responses to disturbance have been observed; for example, those species that rely exclusively on corals for food, shelter or larval settlement are vulnerable to local extinction [19, 20]. For example, corallivorous butterflyfishes are especially prone to declining in abundance following the loss of live corals [15, 19, 21, 22], as are planktivorous Pomacentridae [19, 23], which become vulnerable to increased predation through a loss of shelter [24]. Such losses have been attributed to reductions in habitat complexity that occur after severe physical disturbances [18, 25], or through the gradual erosion of dead coral skeletons after coral bleaching or *Acanthaster planci* infestations [26, 27]. Furthermore, different types of habitat disturbance can manifest in the fish community over different time scales. For example, a coral bleaching event that leaves the reef framework intact but leads to the gradual erosion of coral skeletons can take much longer to affect the fish community than a cyclone that causes the immediate loss of structural complexity [25]. The loss of vulnerable branching corals appears likely to influence a greater suite of fish species than more robust corals, due to the greater structural complexity offered by branching species [17, 28]. On the other hand, coral mortality can leave the substratum bare to be rapidly colonised by algae, leading to an increase in food resources for grazers which may boost their numbers [29]. Similarly, the disturbance of loose sediment and rubble can expose burrowing invertebrates, improving foraging success for invertivores. Therefore, quantifying changes to fish communities can be difficult, as some species decline following disturbances, while others may increase [30, 31, 32].

Alterations to fish communities by disturbances can cause reductions in critical ecosystem functions, potentially leading to regime shifts to less desirable dominant benthic assemblage types (e.g. coral to macro-algal dominance). For instance, it is widely recognised that grazing fishes are integral to the prevention of macroalgal proliferation after disturbances [33, 34, 35]. The loss of predators may mediate trophic cascades, leading to changes in the relative abundance of lower trophic groups [36], although this has been more difficult to detect in complex systems such as coral reefs [37]. For large groups of fishes, such as planktivores, omnivores, macroinvertivores and territorial farmers, the poor state of our current knowledge about their functional roles means that the consequences of their decline for ecological functioning are unpredictable.

**Fig 1:**
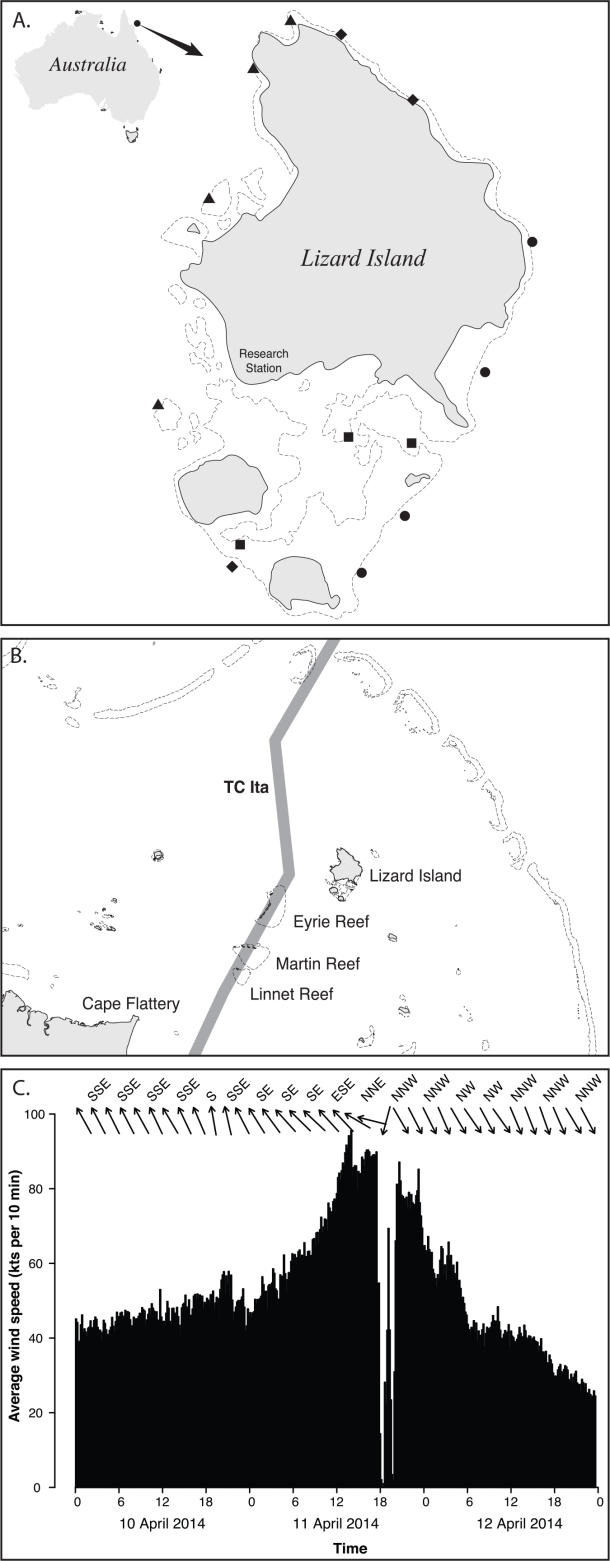
Lizard Island and Severe Tropical Cyclone Ita. A. Lizard Island with positions of surveyed sites. Symbols identify the degree of exposure at each site. Diamonds: Oblique; squares: Lagoon; triangles: Sheltered; circles: Exposed. B. Track of Tropical Cyclone (TC) Ita (grey line) in relation to Lizard Island. C. Wind speed and direction throughout the course of Cyclone Ita, measured by IMOS at Lizard Island. The x-axis includes the date and time, the y-axis is wind speed (knots), and wind direction is depicted with arrows across the top of the chart.

The Great Barrier Reef (GBR) has recently undergone a series of very intense (Category 5) tropical cyclones that have contributed significantly to an overall decline in coral cover of almost 50% over the past 27 years [38]. In April 2014 Tropical Cyclone Ita carved a path across the northern Great Barrier Reef, tracking almost directly over Lizard Island (14°39.873S, 145°26.715E), a high granitic island lying halfway between the mainland and the edge of the continental shelf (Fig 1). Three years prior to this, an extensive study linking fish and coral species diversity was undertaken at 14 sites around Lizard Island, enabling the examination of the underlying effects of different exposure regimes on changes in the fish communities of Lizard Island coral reefs following Cyclone Ita. In particular, we ask the following questions:

- How did coral cover, fish density, biomass, species richness and assemblage structure change following Cyclone Ita, and did changes vary among sites with different exposure regimes?
- Did certain species suffer greater declines than others, and are there consistent patterns in their trophic affiliations?
- How well did the results at broad resolutions (e.g. total abundance) compare with those done at finer resolution, such as species level analyses?

We show that the degree of change measured at coarse levels masked high species-level turnover, and that fish community changes after the cyclone were strongly affected by wave exposure and depth.

## Materials and Methods

Coral and fish communities were surveyed at fourteen sites at Lizard Island on the northern Great Barrier Reef (14°39.873S, 145°26.715E) in September 2011 and again in January 2015 (Fig 1), 10 months after Cyclone Ita crossed the island. This research was conducted at Lizard Island Research Station, a facility of the Australian Museum. Permits to conduct the research are granted by the Great Barrier Reef Marine Park Authority; relevant permits are held by the Museum. The field studies did not involve protected or endangered species, and no ethics approvals were required.

The sites were chosen to maximise the diversity of habitats and exposure regimes found at Lizard Island, including protected fringing and patch reefs, lagoonal habitats, reef passes and steep reefs slopes exposed to the prevailing weather. Sites were classified by exposure to the prevailing sou-easterly trade winds and included four sheltered (northwest facing), three lagoon, four oblique (northeast and southwest facing) and three exposed (southeast facing) sites (Fig 1).

Coral and reef fish surveys were conducted concurrently at two depths per site; shallow (3-5m) and deep (8-10m). The first observer (alternately MJE and DMC in 2011; DMC in 2015) recorded the abundance and size (total length to the nearest cm) of all diurnal, non-cryptic reef fish species along three 50 m transects (5 m wide belt for larger, mobile fishes and 1 m wide belt for smaller, site-attached fishes). Fish density was expressed as individuals per 1000 m^2^; species richness was used as a measure of diversity. Biomass was calculated according to length-weight relationships listed in Kulbicki et al. [39] and expressed as kg 1000m^-2^. Comparisons of average length estimates indicated that for 97 out of 113 fish species, there were no differences between the estimates between the two observers. Where there were differences, we detected no systematic biases that should influence the inference of a change in biomass before and after the storm. MJE’s size estimates were higher for *Acanthurus blochii*, *Chaetodon baronessa*, *Chaetodon ephippium* and *Plectropomus leopardus*; DMC’s were higher for *Diagramma pictum* and *Naso unicornis* (S1 Fig). The second observer (ZTR) recorded live hard coral cover at 100 uniformly distributed points per transect.

### Statistical analyses

To examine the status of fish communities 10 months after Cyclone Ita, and how these varied among sites of differing exposure and depth, we used Bayesian hierarchical mixed models, fitted separately for hard coral cover, total fish species richness, the density and biomass of the total fish assemblage and individual families, and the density, biomass and length of individual species. Models had the fixed factors of Exposure, Depth and Time (before and after Cyclone Ita). Site and transect were treated as random factors to account for spatial and temporal autocorrelation associated with monitoring the same sites repeatedly. Most fish density and biomass variables were modelled against a lognormal distribution, although the density and biomass of some families contained a large number of zeroes and were modelled against a zero-inflated lognormal distribution. To visualise size changes, length-frequency histograms were produced for the most abundant species that displayed discordant density-biomass shifts between 2011 and 2015.

To then compare how well high level taxonomic summary statistics performed, we compared the results of assemblage and family level analysis to models performed for individual fish species which occurred in high enough abundance to enable models to converge. Models were fitted to the density of 72 species and biomass of 65 species, with Exposure, Depth and Time fitted as fixed factors whilst Site and Transect were fitted as random factors. All models were fitted through Just Another Gibbs Sampler (JAGS) via the R2JAGS package in R and used non-informative flat priors and the posterior distributions were derived from three Markov chain Monte Carlo (MCMC) samplers of 500,000 iteration after a burn-in of 250,000 and a thinning of 100. Model convergence and mixing of Markov chains was assessed visually from trace plots and autocorrelations of chains was always less than 0.2. Inferences about temporal changes were based on Bayesian 95% uncertainty interval of Highest Posterior Density (HPD) cell medians predicted from posterior distributions of model parameters. Specific post hoc contrasts were examined including the differences in response variables before and after Cyclone Ita among exposures. To explore the direct relationships between the fish community at three different resolutions (summary metrics, family level and species level) and changes in coral cover, we regressed the 2011 to 2015 change in fish biomass and density on the change in hard coral cover.

To visualise changes in fish communities before and after Cyclone Ita among sites of differing exposure to the prevailing weather, we performed a constrained Redundancy Analysis (RDA) based on fish biomass using the vegan package in R. Fish biomass was transformed prior to analysis using the Hellinger metric [40] to reduce the influence of highly abundant taxa. Constrained RDA allows the removal of the effect of some conditioning variables, in this case exposure, and enables the partitioning of variation among constrained and ‘cleansed’ variables (in this case time before or after the cyclone). To determine the relative contribution of the fish species to the final RDA solution, each variable was projected onto the ordination space. Vectors were calculated using the partial regression coefficients of the original variables within the two dimensions of the RDA, and the lengths of the vectors were set proportional to the squared multiple correlation coefficient.

## Results

**Fig 2:**
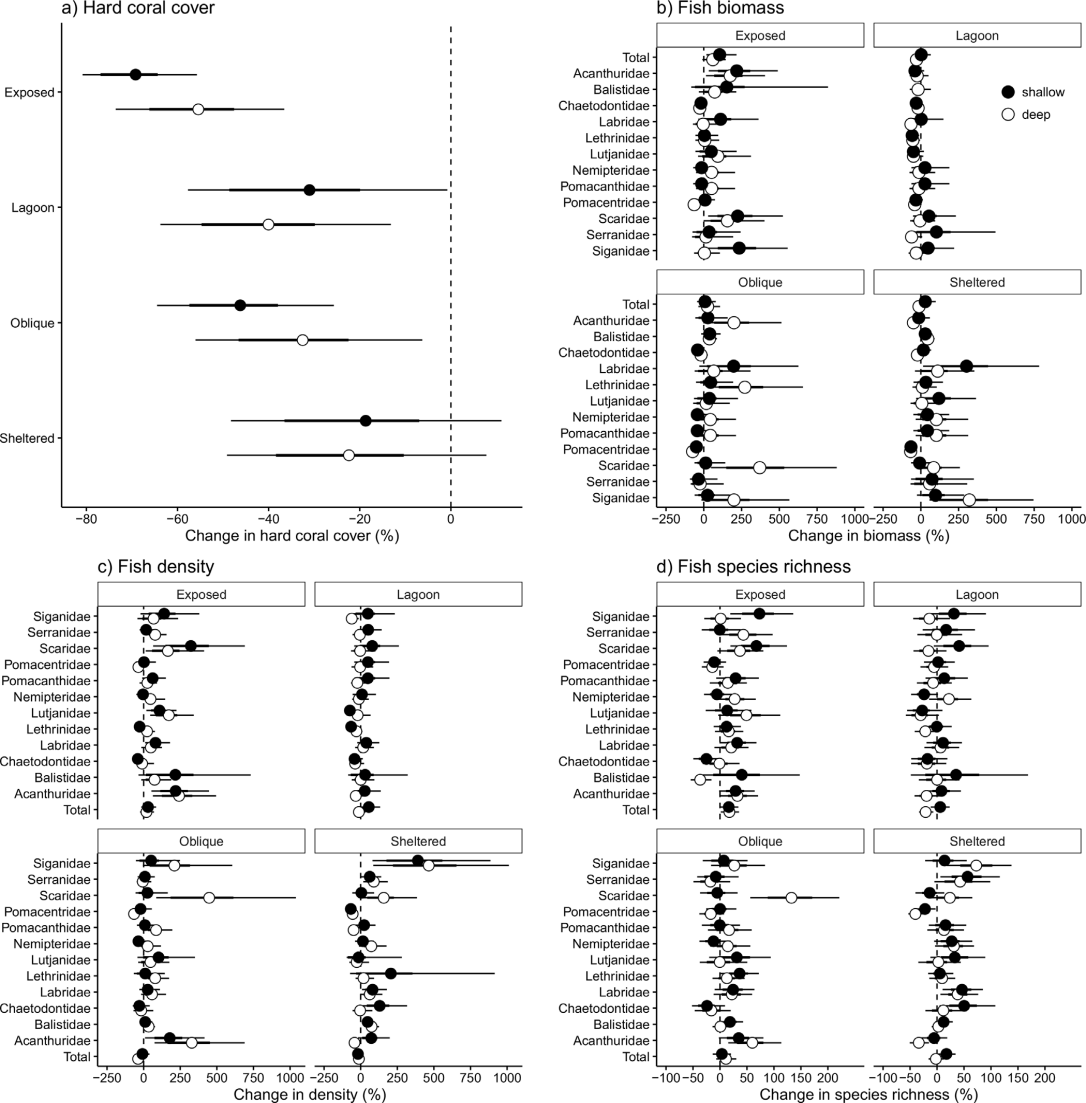
Changes in coral cover, total fish metrics and individuals fish families between pre- and post- Cyclone Ita surveys. Closed circles: shallow; open circles: deep. A. % live hard coral cover; B. fish biomass (kg 1000m^-2^); C. fish density (individuals 1000m^-2^); D. fish species richness. Data are modelled median differences between 2011 and 2015 surveys. Error bars are 50% (thick lines) and 95% (thin lines) uncertainty intervals (UIs). Data were modelled using a Bayesian hierarchical linear mixed model, and differences are expressed as a percentage of the pre-cyclone value. Statistical significance is inferred where 95% UIs do not intersect zero.

Cyclone Ita caused extensive damage to the fringing coral reefs around Lizard Island. Despite high variability among sites, coral cover was lost across all exposure categories except sheltered sites, with the largest decline at exposed sites (Fig 2A). There was no difference in the decline of hard coral cover between depths in any of the four exposures. There was strong evidence that the decline at deep exposed sites was greater than the decline at shallow lagoon sites, deep oblique sites and both shallow and deep sheltered sites. However, there was much weaker evidence for differences between any of the other sites (Fig 2A).

There was no evidence of an overall change in the total biomass of reef fishes at either depth in sites of any exposure to the prevailing weather, except for a 50% increase in total biomass at deep exposed sites (Fig 2B). Similarly, there were few changes in either total density or total species richness of reef fishes, except for small declines in density at deep oblique sites and species richness at deep lagoon sites, and small increases in species richness at both shallow and deep exposed sites (Fig 2C and 2D). However, large significant changes became apparent when examining individual fish taxa (families and species).

The density and biomass of different fish families did not change uniformly across exposure regimes between 2011 and 2015 (Fig 2B and 2C). For example, Acanthurid density increased at exposed and oblique sites, but did not change at sheltered and lagoonal sites. Additionally, there were few differences in the density, biomass or species richness of the majority of fish families with depth (Fig 2B, 2C and 2D). One of the few exceptions was a more than doubling of the species richness of Scaridae at deep oblique sites, with no concomitant change in the shallows (Fig 2D). That notwithstanding, more families increased in density and/or biomass at exposed sites than elsewhere, while a larger number of fish families declined at lagoon sites than in other habitats. Deep lagoonal sites also experienced the only significant overall loss in fish species richness. Overall, more families increased in density and biomass than decreased following cyclone Ita. The greatest number of families increased in density at sites exposed to the prevailing weather (seven including Acanthuridae, Balistidae, Labridae, Lutjanidae, Serranidae, Siganidae and Scaridae), whilst lagoonal sites had no increases. Similarly, exposed sites also had the greatest number of families increasing in biomass. The greatest number of decreases in density occurred in lagoonal sites. Sheltered and oblique sites had both declines and increases, with no consistent patterns among families.

**Fig 3:**
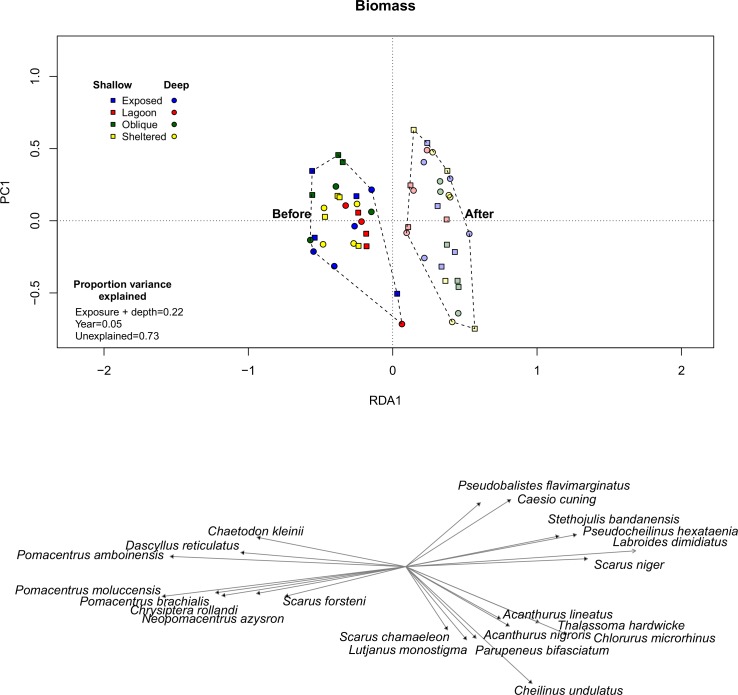
Visualisation of the change in reef fish assemblage structure from before (2011) to after Cyclone Ita (2015) using a constrained Redundancy Analysis. Constrained RDA allows the removal of the effect of some conditioning variables (Exposure) and enables the partitioning of variation attributable to constrained and cleansed variables. RDA1 therefore displays the variation due to the constrained factor (Exposure). To downplay the role of highly abundant species, fish biomass was transformed using the Hellinger metric [40]. Different colours denote wave exposure categories, squares are deep transects and circles are shallow transects. Vectors show fish species driving the greatest proportion of the variability.

Once the variation due to exposure and depth (22% of the total variation) was accounted for in a redundancy analysis, changes were apparent in fish assemblage structure after Cyclone Ita, although this accounted for only 5% of the total variation (Fig 3). There were no clear generalities in the direction of assemblage change among depth and exposure regimes. Most assemblage changes appear to be driven by a loss of damselfish species, most notably *Pomacentrus amboinensis*, *Pomacentrus brachialis*, *Pomacentrus chrysurus*, *Pomacentrus moluccensis and Neopomacentrus azysron* and increases in some herbivorous fishes like *Chlorurus microrhinus*, *Acanthurus nigrofuscus* and *Acanthurus lineatus*, particularly in shallow sites exposed and oblique to the prevailing weather (Fig 3).

Of the total species pool (beyond those chosen for modelling), 44 species were recorded in 2011 but not in 2015, although 43 of these were rare in 2011 with densities <5 per 1000m^2^; 38 novel species were seen in 2015 that had not been recorded in 2011. Of the species present in both years, 37 species (18%) declined more than 50% in density, and 54 species (26%) declined more than 50% in biomass. Conversely, 100 species (74% of species present in both years) increased by at least 50% in density, and 82 species (75%) increased by at least 50% in biomass. Species that declined in biomass and density were predominantly planktivores, followed by mobile invertebrate feeders and piscivores; species that increased were dominated by herbivores and omnivores.

**Fig 4:**
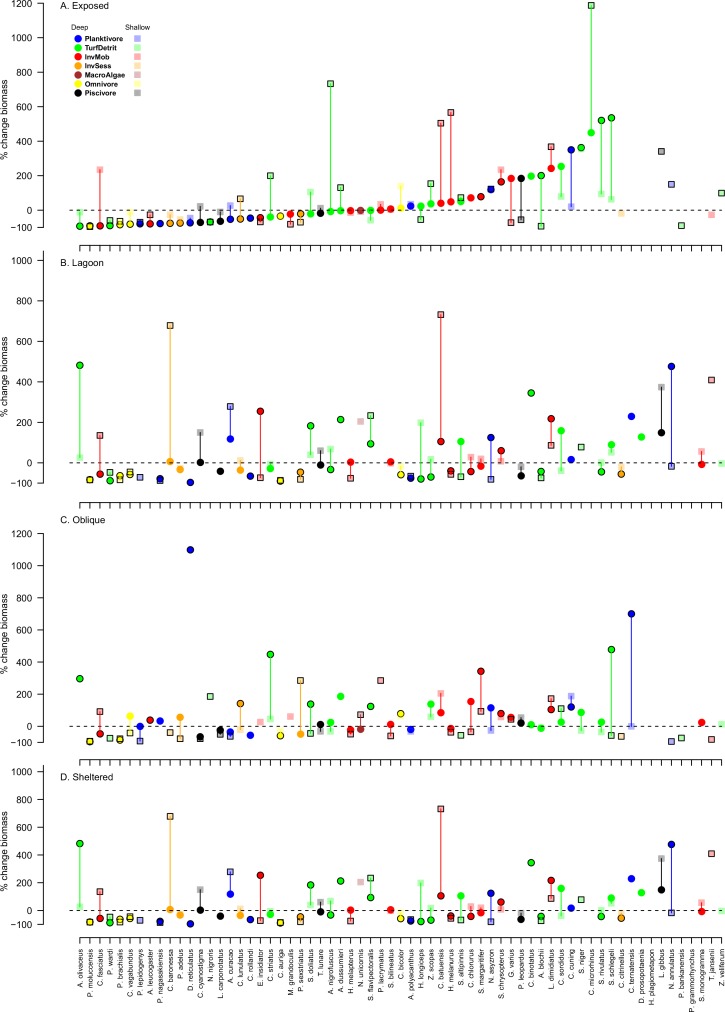
**Average percentage change in the biomass of individual fish species between 2011 and 2015** for A. exposed, B. lagoon, C. oblique and D. sheltered sites of Lizard Island. Fish species were only included in analyses if there were at least 10 individuals in both years. The y axis is the percent change in biomass. Colours represent trophic affiliations: blue = planktivores, orange = sessile invertebrate feeders, white = omnivores (feeding on both plant and animal matter), green = turf and detritus feeders, red = mobile invertebrate feeders, black = piscivores and dark red = macroalgal feeders. Symbols with black outlined represent species for which biomass changed significantly at that depth-exposure combination. Vertical lines link deep and shallow symbols for each species and are for ease of observation.

There were substantial changes in the biomass of individual species of reef fishes, although there were very few consistent changes among depths and exposure categories following Cyclone Ita. Four species, *Chrysiptera rollandi*, *Pomacentrus wardi*, *Pomacentrus moluccensis* and *Pomacentrus brachialis*, declined in biomass in all depth and exposure combinations (Fig 4). Additionally, a number of species declined in most of the depth and exposure combinations, and these tended to be intimately associated with hard coral cover, like the butterflyfishes *Chaetodon auriga* and *Chaetodon vagabundus* (Fig 4). Between 26% and 35% of the species pool available for analysis declined in biomass in each depth by exposure location, except for Oblique sites, which had both the greatest and smallest proportional species decline (40% in the shallows and 13% in deeper areas). Species undergoing declines in biomass were typically planktivores, omnivores or turf and detritus feeders, although the proportions varied among depth and exposure combinations.

Similarly, no species consistently increased in biomass at all locations following Cyclone Ita. There was also no clear depth or exposure related trend in the number of species that increased in biomass, which ranged from 16% (Exposed deep and Oblique shallow) to 31% (Oblique deep) of the species pool. Species that increased in biomass tended to be turf and detritus feeders, notably the parrotfishes *Scarus niger*, *Scarus schlegeli*, the surgeonfish *Acanthurus nigrofuscus* and the rabbitfish *Siganus doliatus*. However, a few mobile invertebrate feeders also increased in biomass and included *Coris batuensis*, *Scolopsis margaritifer* and *Labroides dimidiatus* (Fig 4).

**Fig 5:**
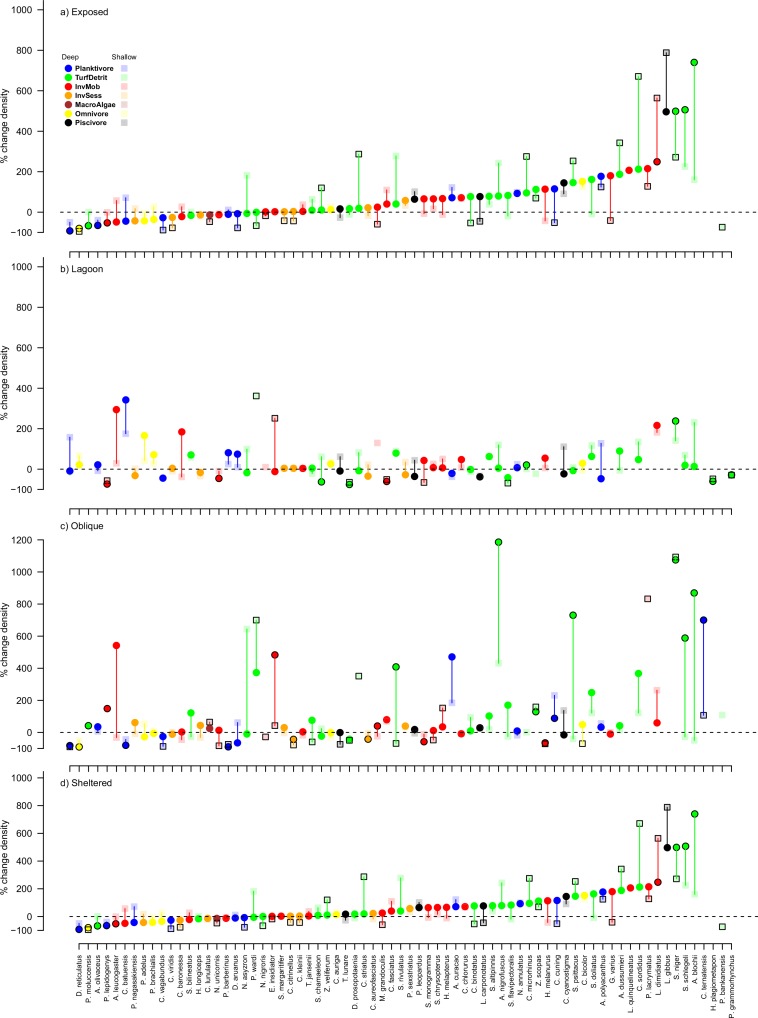
**Average percentage change in the density of individual fish species between 2011 and 2015** for A. exposed, B. lagoon, C. oblique and D. sheltered sites of Lizard Island. Fish species were only included in analyses if there were at least 10 individuals in both years. The y axis is the percent change in density. Colours represent trophic affiliations: blue = planktivores, orange = sessile invertebrate feeders, white = omnivores (feeding on both plant and animal matter), green = turf and detritus feeders, red = mobile invertebrate feeders, black = piscivores and dark red = macroalgal feeders. Symbols with black outlined represent species for which biomass changed significantly at that depth-exposure combination. Vertical lines link deep and shallow symbols for each species and are for ease of observation.

Changes in density were similar to changes in biomass, in that species that declined tended to be planktivores and omnivores, and increases were more common among turf and detritus feeders (Fig 5). No species declined in density at all exposure and depth combinations, but between 1 and 32% of species declined in each exposure and depth combination. There was a tendency towards greater declines in deep habitats of relatively exposed sites, and greater declines in shallow habitats of relatively sheltered sites. Notable declines in density occurred in most exposure and depth combinations for *Dascyllus reticulatus*, *Chromis viridis* and *Pomacentrus moluccensis*.

Two species, *Scarus niger* and *Labroides dimidiatus*, increased in density in all exposure and depth combinations (Fig 5). Density increases were recorded for a larger range of species than density declines, with between 22% and 55% of species increasing in density in at least one depth and exposure combination. Species that increased tended to be grazing fishes.

**Fig 6:**
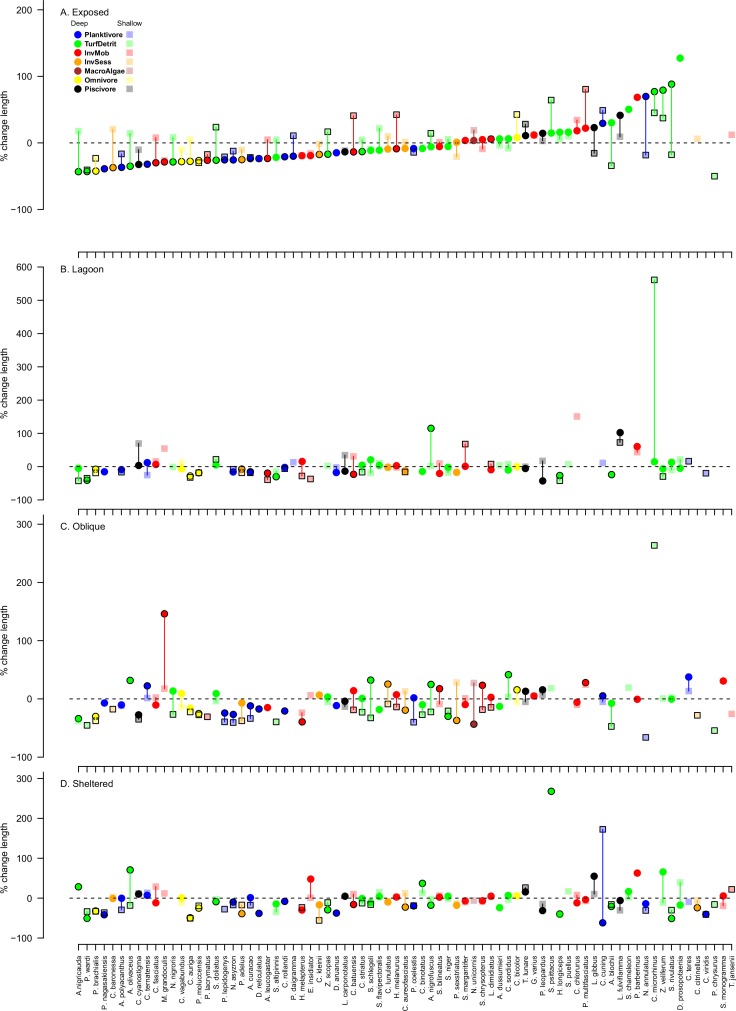
**Average percentage change in the total length (cm) of individual fish species between 2011 and 2015** for A. exposed, B. lagoon, C. oblique and D. sheltered sites of Lizard Island. Fish species were only included in analyses if there were at least 10 individuals in both years. The y axis is the percent change in fish total length. Colours represent trophic affiliations: blue = planktivores, orange = sessile invertebrate feeders, white = omnivores (feeding on both plant and animal matter), green = turf and detritus feeders, red = mobile invertebrate feeders, black = piscivores and dark red = macroalgal feeders. Symbols with black outlined represent species for which total length changed significantly at that depth-exposure combination. Vertical lines link deep and shallow symbols for each species are for ease of observation.

**Fig 7:**
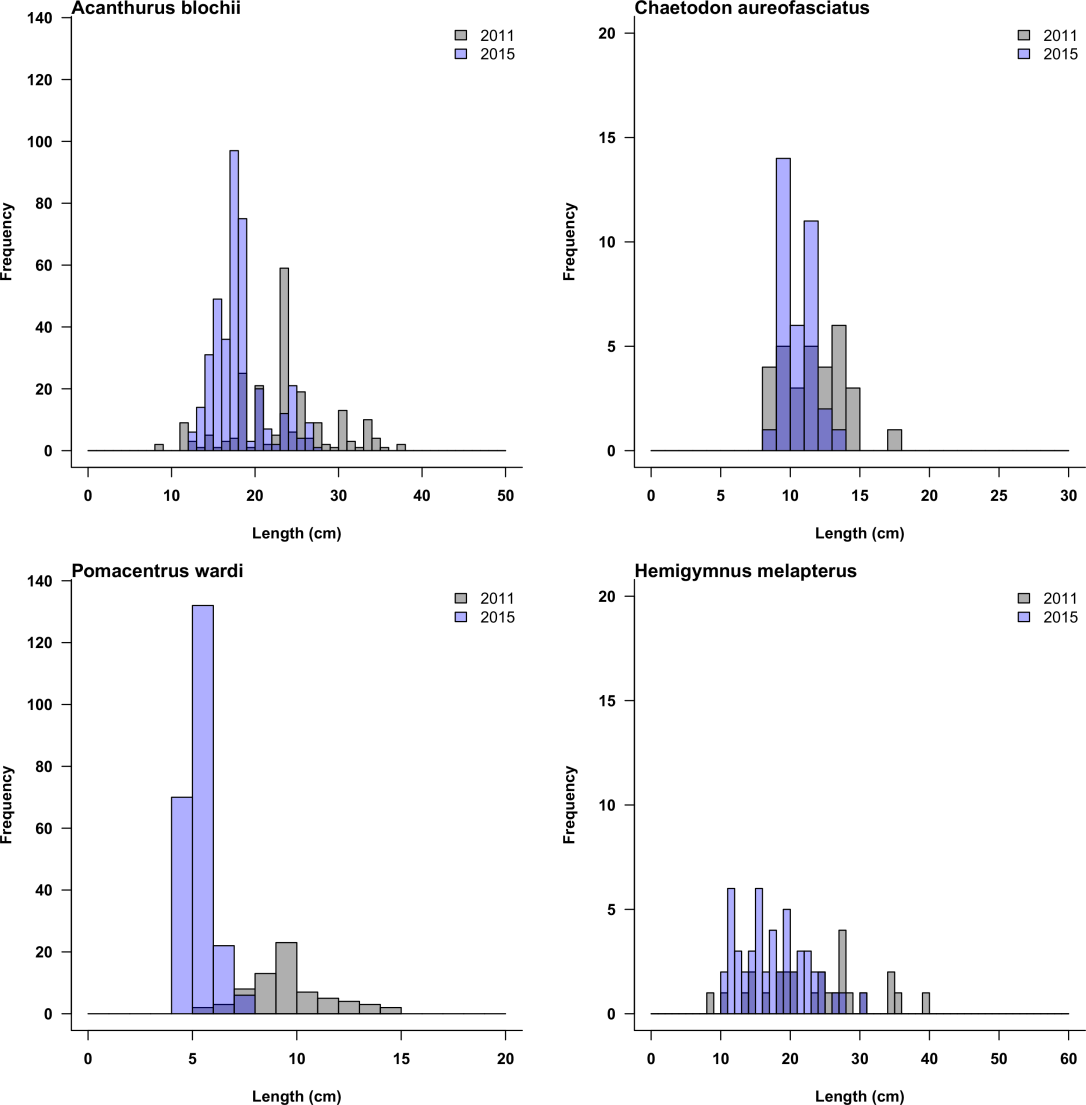
Examples of the length-frequency distribution of four species that increased in abundance, but declined in biomass from before (2011) to after Cyclone Ita (2015).

Changes in total length also varied with exposure and depth, more species declining in length (between 17 and 38%) than increasing (between 1 and 17%) across all exposures and depths (Fig 6). Typically, the average length of omnivorous and planktivorous pomacentrids (e.g. *Pomacentrus brachialis*, *Pomacentrus moluccensis*, *Pomacentrus nagasakiensis*, *Neopomacentrus azysron*) and corallivorous chaetodontids (e.g. *Chaetodon baronessa*, *Chaetodon vagabundus*, *Chaetodon auriga*) declined after Cyclone Ita, while the functional affiliation of species that increased in length appeared more random. Some species declined in length at one depth or exposure, whilst increasing elsewhere; for instance, *Caesio cuning* was larger in shallow habitats of the sheltered sites, but smaller in deeper habitats, whilst *Acanthurus olivaceus* displayed the opposite pattern. Interestingly, 27 species declined in biomass but increased in density (Table 1), and many of these species declined in average length. Importantly, these species also displayed a loss of larger size classes, with a concomitant dominance of small size classes (Fig 7).

**Table 1:** Species that declined in biomass but increased in abundance between 2011 and 2015, including their family and trophic affiliations. Mean total length (TL, in cm) is presented before and after Cyclone Ita, +/- 1 SE (shown in brackets), along with the number of individuals (N) recorded in each survey.

Family	Trophic group	Species	TL before	N	TL after	N
**Acanthuridae**	Grazer—detritivore	*A*. *blochii*	23.7 (0.4)	216	19 (0.2)	387
	Algal cropper	*Zebrasoma veliferum*	17.8 (1)	38	16.5 (0.6)	42
**Balistidae**	Omnivore	*Balistapus undulatus*	17.1 (2.4)	11	16.6 (1.3)	13
**Chaetodontidae**	Omnivore	*Chaetodon auriga*	18.6 (0.8)	58	12.9 (0.3)	70
	Obligate corallivore	*C*. *aureofasciatus*	12.2 (0.4)	31	11.2 (0.2)	35
	Facultative corallivore	*C*. *ulietensis*	14.2 (0.8)	10	13.1 (0.7)	13
	Microinvertivore	*Heniochus monoceros*	19.7 (2.9)	3	14.5 (0.4)	3
**Haemulidae**	Carnivore	*Plectorhinchus chaetodontoides*	52.3 (7.7)	6	41.9 (2.9)	7
	Carnivore	*P*. *flavomaculatus*	43.3 (7.3)	3	36.6 (2.9)	5
**Labridae**	Macroinvertivore	*Cheilinus chlorurus*	17.7 (1.1)	40	16.8 (0.6)	52
	Macroinvertivore	*Coris aygula*	34 (0)	1	27 (0)	1
	Macroinvertivore	*Halichoeres margaritaicus*	10.2 (1.5)	5	9.3 (0.3)	7
	Macroinvertivore	*Hemigymnus melapterus*	23.9 (1.5)	27	19.1 (0.8)	52
**Labridae (Scarinae)**	Scraper	*Scarus dimidiatus*	30 (0.9)	6	23.5 (1.9)	10
	Scraper	*S*. *flavipectoralis*	20 (0.5)	138	20.3 (0.4)	149
**Lutjanidae**	Piscivore	*Lutjanus bohar*	37.1 (6.7)	11	27.4 (2.4)	18
**Pomacanthidae**	Spongivore	*Pomacanthus semicirculatus*	31 (4)	2	19.3 (3.9)	3
**Pomacentridae**	Planktivore	*Amblyglyphidodon curacao*	9.9 (0.1)	429	8.4 (0.1)	592
	Planktivore	*A*. *leucogaster*	11.1 (0.3)	66	10.1 (0.2)	69
	Planktivore	*Chromis margaritifer*	7 (0)	1	4.3 (0.3)	4
	Planktivore	*C*. *viridis*	6.7 (0.1)	317	4.9 (0.1)	718
	Omnivore	*Neoglyphidodon melas*	11.5 (1.8)	6	7.3 (0.5)	40
	Farming grazer	*Pomacentrus wardi*	9.9 (0.3)	71	4.6 (0.1)	528
**Serranidae**	Carnivore	*Diploprion bifasciatum*	18.3 (2.2)	4	16.3 (0.9)	4
	Carnivore	*Epinephelus fuscoguttatus*	90 (0)	1	50.5 (0.5)	2
	Carnivore	*E*. *maculatus*	28.5 (2.7)	8	21.1 (2.7)	11

Increasing the taxonomic resolution of the study revealed changes at the species level that were hidden at the coarser whole-assemblage resolution and the family level (Table 2). At the whole-assemblage level, density did not change at any of the depth-exposure combinations, biomass increased only at shallow exposed sites and species richness declined only at shallow lagoon sites. At the family level, there was no change in density in 58–92% of families (depending on the depth-exposure combination), no change in biomass for 67–92% of families and no change in species richness in 67–100% of families. However at finer taxonomic resolution, a far lower percentage of species remained stable, with greater proportions of species showing some degree of change. Similar numbers of species increased in density and biomass as decreased or remained stable in most depth and exposure combinations. In most depth and exposure combinations, only 20–30% of species showed no change in density and only between 8 and 27% of species showed no change in biomass (except deep exposed sites, where 86% of species did not change in density). More than half of the species available for analysis at each depth and exposure combination declined or increased in the wake of the cyclone.

**Table 2:** Stability and change in reef fish density, biomass and species richness at different taxonomic resolutions, partitioned by exposure and depth. Numerals in each of the depth x exposure regimes show the number of families or species that increased (↑), decreased (↓) and remained stable (-).

Taxonomic resolution	Metric	Change	Exposed		Sheltered		Oblique		Lagoon	
			Deep	Shallow	Deep	Shallow	Deep	Shallow	Deep	Shallow
**Total**	density	↑								
		↓								
		-	1	1	1	1	1	1	1	1
	biomass	↑		1						
		↓								
		-	1		1	1	1	1	1	1
	species richness	↑								
		↓								1
		-	1	1	1	1	1	1	1	
**Family**	density	↑	3	4	3	3	2	1		
		↓			2	1	1		1	2
		-	9	8	7	8	9	11	11	10
	biomass	↑	1	4	2	1	2			
		↓	1		2	1	1	2	1	1
		-	10	8	8	10	9	10	11	11
	species richness	↑	2		3	2		2		
		↓		1		2				
		-	10	11	9	8	12	10	12	12
**Species**	density	↑	5	12	18	25	35	23	23	31
		↓	5	14	18	4	13	22	18	13
		-	61	50	27	10	15	12	19	12
	biomass	↑	21	26	18	19	28	18	17	21
		↓	22	21	25	14	9	28	26	17
		-	11	7	9	12	13	4	8	10

The relationship between fish taxa and hard coral cover also varied, with weak correlations at the level of summary metrics and slightly stronger relationships at higher taxonomic resolutions (Table 3). Correlations between the 2011–2015 change in percent cover of hard coral and the change in density, biomass and species richness of fishes were generally weak, with a highest R^2^ value of 0.3978. The strongest relationships (R^2^ > 0.25) were found for the density of *Acanthurus dussumieri*, *A*. *olivaceus*, *Scarus frenatus* (negative), *Chaetodon baronessa* and *Neoglyphidodon melas* (positive) and the biomass of *A*. *dussumieri*, *Parupeneus multifasciatus* and *Siganus corallinus* (negative), and *C*. *baronessa* and *Chromis ternatensis* (positive). Generally, the change in grazing fishes tended to be negatively correlated with changes in coral cover, whilst changes in corallivores and small planktivores tended to be positively associated.

**Table 3:** R^2^ regression values of changes in the fish community between 2011 and 2015 vs. changes in coral cover. Regressions were performed on the site-depth averages of changes in density and biomass. All summary metrics are shown. For family and species level regressions, only taxa with an R^2^ of at least 0.1 are given. Significant relationships are identified with a * for p < 0.05, ** for p < 0.01, and *** for p < 0.001. See Supplementary material for corresponding Figures (S2 Fig).

Taxonomic resolution	Metric	Taxon	R^2^	Positive / negative correlation
Summary	Density		0.0049	Negative
	Biomass		0.0879	Negative
	Species richness		0.0212	Positive
Family	Density	Acanthuridae*	0.1901	Negative
		Chaetodontidae*	0.2058	Positive
		Haemulidae	0.1371	Negative
	Biomass	Acanthuridae*	0.2217	Negative
Species	Density	Acanthurus dussumieri**	0.3336	Negative
		*Acanthurus nigricauda*	0.1259	Negative
		Acanthurus olivaceus**	0.256	Negative
		*Naso annulatus*	0.1088	Negative
		*Zebrasoma veliferum*	0.1383	Negative
		Chaetodon baronessa**	0.337	Positive
		*Chaetodon rainfordi*	0.1027	Positive
		*Chaetodon ulietensis*	0.131	Positive
		Cheilinus chlorurus*	0.1564	Negative
		*Choerodon fasciatus*	0.1061	Positive
		*Hemigymnus melapterus*	0.121	Positive
		*Pseudocheilinus hexataenia*	0.112	Positive
		Lethrinus atkinsoni*	0.1679	Negative
		Lutjanus bohar*	0.1964	Negative
		*Lutjanus carponotatus*	0.1204	Positive
		Lutjanus gibbus*	0.1467	Negative
		*Lutjanus quinquelineatus*	0.1033	Positive
		*Pomacanthus sexstriatus*	0.1005	Positive
		Chromis ternatensis*	0.1891	Positive
		*Hemiglyphidodon plagiometapon*	0.118	Positive
		Neoglyphidodon melas**	0.2845	Positive
		*Pomacentrus amboinensis*	0.1189	Negative
		Pomacentrus bankanensis*	0.1684	Positive
		Scarus frenatus***	0.3978	Negative
		*Cephalopholis argus*	0.1329	Positive
		Siganus corallinus*	0.226	Negative
		Siganus doliatus*	0.1601	Positive
	Biomass	Acanthurus dussumieri**	0.2891	Negative
		*Acanthurus nigrofuscus*	0.1167	Negative
		*Ctenochaetus striatus*	0.1055	Negative
		*Naso unicornis*	0.1178	Negative
		Zebrasoma veliferum*	0.1422	Negative
		Chaetodon baronessa**	0.2434	Positive
		Cheilinus chlorurus*	0.2191	Negative
		*Pseudocheilinus hexataenia*	0.1033	Positive
		Lethrinus atkinsoni*	0.1693	Negative
		*Monotaxis grandoculis*	0.1148	Positive
		*Lutjanus gibbus*	0.1098	Negative
		Lutjanus quinquelineatus*	0.1432	Positive
		Parupeneus multifasciatus**	0.3012	Negative
		*Pomacanthus sexstriatus*	0.1027	Positive
		Amblyglyphidodon curacao*	0.2059	Positive
		*Amblyglyphidodon leucogaster*	0.109	Positive
		Chromis ternatensis**	0.2506	Positive
		*Neoglyphidodon melas*	0.112	Positive
		Pomacentrus adelus*	0.1864	Negative
		*Pomacentrus amboinensis*	0.1188	Negative
		Pomacentrus nagasakiensis*	0.179	Negative
		*Stegastes apicalis*	0.1195	Negative
		*Scarus frenatus*	0.1098	Negative
		*Scarus niger*	0.114	Negative
		*Scarus psittacus*	0.1129	Negative
		*Cephalopholis argus*	0.116	Positive
		Siganus corallinus**	0.2329	Negative
		*Siganus doliatus*	0.1026	Positive
		Zanclus cornutus*	0.1467	Negative

## Discussion

Cyclone Ita was a significant disturbance event that was followed by considerable changes in coral cover and the reef fish assemblage of Lizard Island, however few of these changes were consistent across depth and wave exposure regimes. This reflects the range of community types and their different susceptibilities to storm swell across a range of exposures to the prevailing weather at Lizard Island, coupled with the change in wind direction and duration of the impacts from heavy swell as the storm passed the island. Long-term data on coral cover, fish abundance and fish species richness shows a history of relative stability in the region of Lizard Island, especially between 2011 and 2015, with the only recent major decline coinciding with Cyclone Ita (S3 Fig). While hard coral cover was reduced at most sites, changes to fish assemblages were dependent on taxonomic resolution. Coarse metrics such as total fish density, biomass or species richness presented inherent stability and resistance to the effects of disturbance, however drilling down to finer taxonomic scales such as family or species level revealed substantial changes, which in turn varied among depth and wave exposure regimes. The disparity between results of different resolution is most probably due to substantial species-level turnover, which is smoothed over or averaged when viewed at coarser scales. The changes documented at fine taxonomic scales are likely to affect ecological functioning, particularly as groups of fishes performing certain functional roles appeared to consistently be ‘winners’ (e.g. herbivorous fishes) and ‘losers’ (e.g. fishes closely associated with hard coral cover such as planktivores and corallivores). Interestingly, some species underwent reductions in biomass through the loss of larger sized individuals, but increased in density. Length frequency analyses revealed substantial potential for recovery of these species, as there had been an accumulation of numbers in the smallest sizes classes, no doubt the result of recruitment since the storm. Annual monitoring of these fish communities will be required to discover their natural variability; this will give future studies more power to detect changes caused by specific events.

Stability in coarse metrics may be attributable to the retention of enough structural complexity and microhabitat diversity at the reef-scape level to maintain overall fish density, biomass and species richness at pre-disturbance levels, especially as it is now becoming clear that the relationship between habitat complexity and fishes is stronger than the relationship between fish assemblages and benthic biota such as corals [15, 18, 25, 41]. In fact, the relationships between changes in live coral and the fish community were weak at a coarse resolution. Fish of different size, mobility, dietary preferences and feeding mode cue to different habitat characteristics, from large-scale “reefscapes” to individual coral colonies, microhabitats or hole sizes [41, 42, 43, 44, 45]. It follows that if this underlying structure remains intact, the overall abundance and diversity of reef fishes is likely to remain resistant to disturbance-induced coral mortality. Another explanation for stability of coarse metrics may relate to short term impacts and rapid recovery. Rapid change is possible in coral reef fish communities, as evidenced by changes in target fish abundance and biomass within two years after the establishment of MPAs [46]. Our results show both declines and increases in the density and biomass of individual species, and indicate a number of species underwent losses in biomass but increases in density, reflecting a larger number of smaller individuals corresponding to a recruitment pulse sometime after the cyclone. Alternatively, fish may have moved into deeper water to avoid the worst of the storm [5], and then moved back once conditions had moderated; larger fishes have been shown to withstand storms better than smaller ones [47]. A final possibility is that a time lag exists between coral mortality and more pervasive changes in the fish community, likely due to the slow erosion of dead coral skeletons, which provide much of the structural complexity on coral reefs [16, 25, 27, 48]. Despite these alternative explanations being plausible, the results from this study suggest that using coarse metrics masks substantial assemblage-level changes evident at finer taxonomic scales and may result in erroneous conclusions about the impact of cyclones on reef fish assemblages.

The fine scale changes reported here result from differential responses among taxa, whereby there was considerable variation between species in the wake of the cyclone among different depth and wave exposure regimes. At the more extreme end of the response continuum, numerous species were not recorded following the cyclone despite being counted in 2011. Coupled with the novel species that were recorded only in 2015 and species that changed density and biomass, this means that there will likely be considerable impacts on ecological functioning. When species that decline or increase consistently belong to the same functional group, species-level changes will translate into reduced or increased redundancy of that particular functional role. In other words, a loss or decline of multiple species from a functional group will erode the capacity for that role to be performed, while the addition or increase of species will enhance the capacity for a given functional role. Grazing fishes and invertivores tended to benefit in the wake of the cyclone, whilst small planktivores, corallivores and farming damselfishes declined at most sites. This was corroborated by the relationships with live coral cover, where declines in live coral were correlated with increases in grazers and some invertivores, but declines in corallivores and small planktivorous fishes. Previous studies on disturbance and overfishing are unequivocal about the role of grazers in mediating coral recovery over algal overgrowth after coral mortality [49], but many of these studies come from systems where grazing fishes are fisheries targets (e.g. [33]). Here, the lack of exploitation and the high existing numbers of grazers, especially after the cyclone, indicate that algal overgrowth will be an unlikely scenario at most sites.

Small fish species reliant on live corals were most vulnerable to the disturbance, especially planktivorous damselfishes [5], corallivorous butterflyfishes [22], and territorial species that rely on loose sediment or rubble. Small planktivorous fishes that use branching corals to shelter can be the first to decline and the last to recover after a severe disturbance event [5, 17] and in this study, planktivore species were among the fishes that underwent the most notable changes (both increases and declines). Unlike the well-documented detrimental effects of losing grazing fishes on coral reefs [50], consequences of the loss of small-bodied planktivores are mostly unknown. Planktivores link energy produced in the pelagic environment to reef-based communities by converting planktonic sources of nutrition into secondary productivity utilised by reef-dwelling fishes, and serve as key prey for reef piscivores [51, 52]. However, the high diversity of coral reef fish communities is expected to lead to high functional redundancy within trophic groups, meaning that these roles may well be absorbed by other members of the reef community. Territorial fishes profoundly alter the benthic communities inside their farms [53], but the broader consequences of their demise are also unclear. Current knowledge places territorial farmers both in the role of enhancing primary productivity [54] and coral diversity [55], and that of indicators of reef degradation [44]. Future experimental work is necessary to ascertain what effects, if any, their loss would have on reef processes.

The variation in damage to the benthic assemblage will be a key factor in determining the response of fishes [15, 18], coupled with how closely reliant individual fish species are on the benthos, and the intrinsic abilities of each species to escape or shelter from damaging forces by hiding or moving [6]. Few fish taxa exhibited biomass declines across all depth and wave exposure regimes following the cyclone, and this likely reflects the differential impacts on the benthos across sites of differing wave exposure; sheltered sites generally had much smaller loss of hard coral cover than sites fully exposed to the prevailing weather. However, lagoonal sites were impacted due to the shifting of large amounts of sand onto the reef bases (DC pers. obs.) and the delicate growth form of lagoonal coral communities, which suffered disproportionately from the change in wind direction. Although not reported, the cyclone appeared to have minimal impact on habitat complexity (Richards and Ceccarelli, unpublished data), despite reductions in hard coral cover, as the underlying substrate has high levels of rugosity even without intact coral colonies.

In conclusion, the extent of the change to Lizard Island’s reef fish assemblage varied depending on the scale of taxonomic resolution. Coarse measures such as total abundance or species richness showed little change after the storm, whereas finer scale taxonomic measures revealed substantial alterations to the fish assemblage after the cyclone. This is most likely due to high species level turnover being masked or smoothed over at coarser resolution, which has important implications for conclusions drawn from studies using these coarse metrics. For example, at a coarse scale one may conclude from this dataset that there was little impact on fish assemblages and ecological functioning, when in fact ecological functioning can be profoundly affected by changes at the species level.

The patchiness of the coral damage from Cyclone Ita means that there are healthy sites that may serve as sources of replenishment for degraded sites with similar habitat characteristics. The likely long-term consequences of this disturbance are that some areas will recover, others will remain degraded, and most will fall somewhere in between (ie. partial recovery, usually with some species composition shifts). Prior experience suggests that benthic regime shifts will be mirrored by an altered fish assemblage over longer time scales [25, 49]. Reefs with a better chance of recovery tend to be those with high structural complexity, in deeper water, with low nutrient and sediment loads, and higher densities of grazing fishes and juvenile corals. Finally, this study confirms that in high-complexity, high diversity ecosystems such as coral reefs, species level analyses are essential to adequately capture the consequences of disturbance events.

## Supporting Information

S1 Fig Observer comparisons. Differences in average fish lengths recorded between observers DMC and MJD during the 2011 survey, for taxa with > 10 individuals recorded by either observer. Error bars are 95% uncertainty intervals (UIs). Data were modelled using a Bayesian hierarchical linear mixed model, and differences are expressed as a percentage of the pre-cyclone value. Statistical significance is inferred where 95% UIs do not overlap between the two observers.
(PDF)

S2 Fig Coral cover relationships. Regressions of coarse metrics, family-level and species-level density and biomass on coral cover. Regressions were performed on the site-depth averages of changes in density and biomass. All summary metrics are shown. For family and species level regressions, only taxa with an R^2^ of at least 0.1 are given.
(PDF)

S3 Fig Historical trends for the Lizard Island sector. Long-term trends in % coral cover, total fish abundance and fish species richness on exposed sites of Lizard Island (red line), MacGillivray Reef (green line) and North Direction Reef (blue line), showing relative stability in coral and fish communities in the 10 years before Cyclone Ita.
(PDF)

S1 Table Raw fish and coral cover data collected from 2011 and 2015 surveys. (XLSX)
