# Supplementary figures and images for: Post-Disturbance Stability of Fish Assemblages Measured at Coarse Taxonomic Resolution Masks Change at Finer Scales
Source: PLoS One. 2016 Jun 10;11(6):e0156232. doi: 10.1371/journal.pone.0156232 (PMC4902313; doi:10.1371/journal.pone.0156232)

taxa

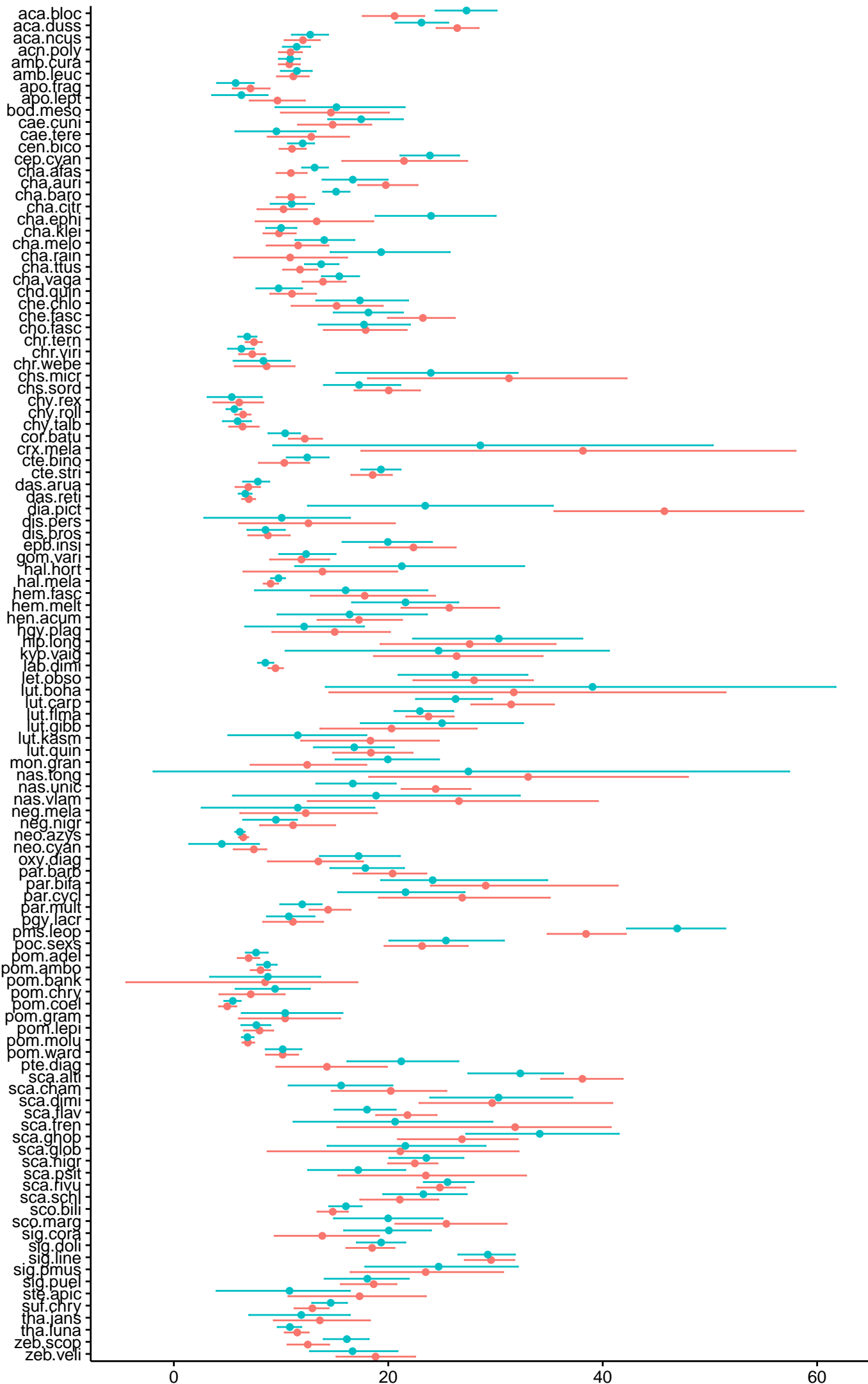

Supplement: S1 Fig — Differences in average fish lengths recorded between observers DMC and MJD during the 2011 survey, for taxa with > 10 individuals recorded by either observer. Error bars are 95% uncertainty intervals (UIs). Data were modelled using a Bayesian hierarchical linear mixed model, and differences are expressed as a percentage of the pre-cyclone value. Statistical significance is inferred where 95% UIs do not overlap between the two observers. (PDF) [file pone.0156232.s001.pdf]

Summary metrics

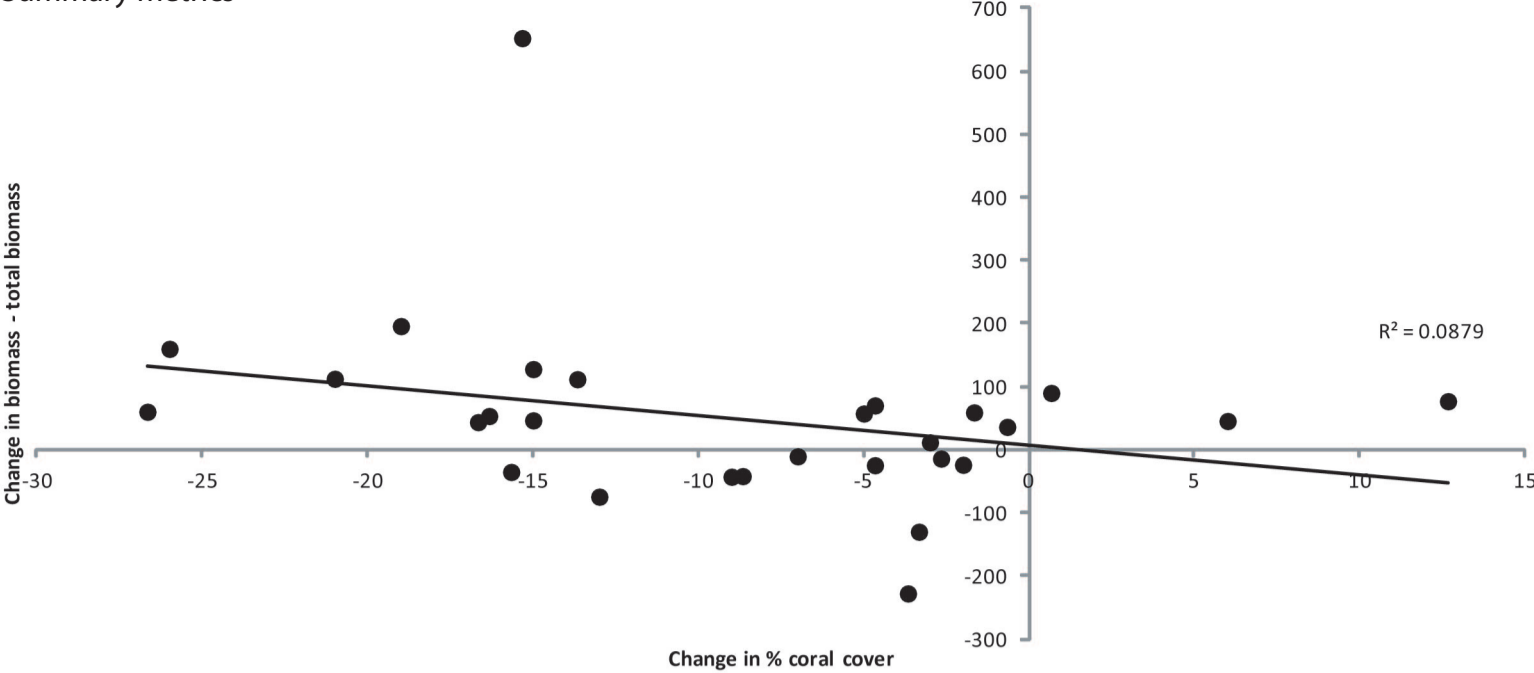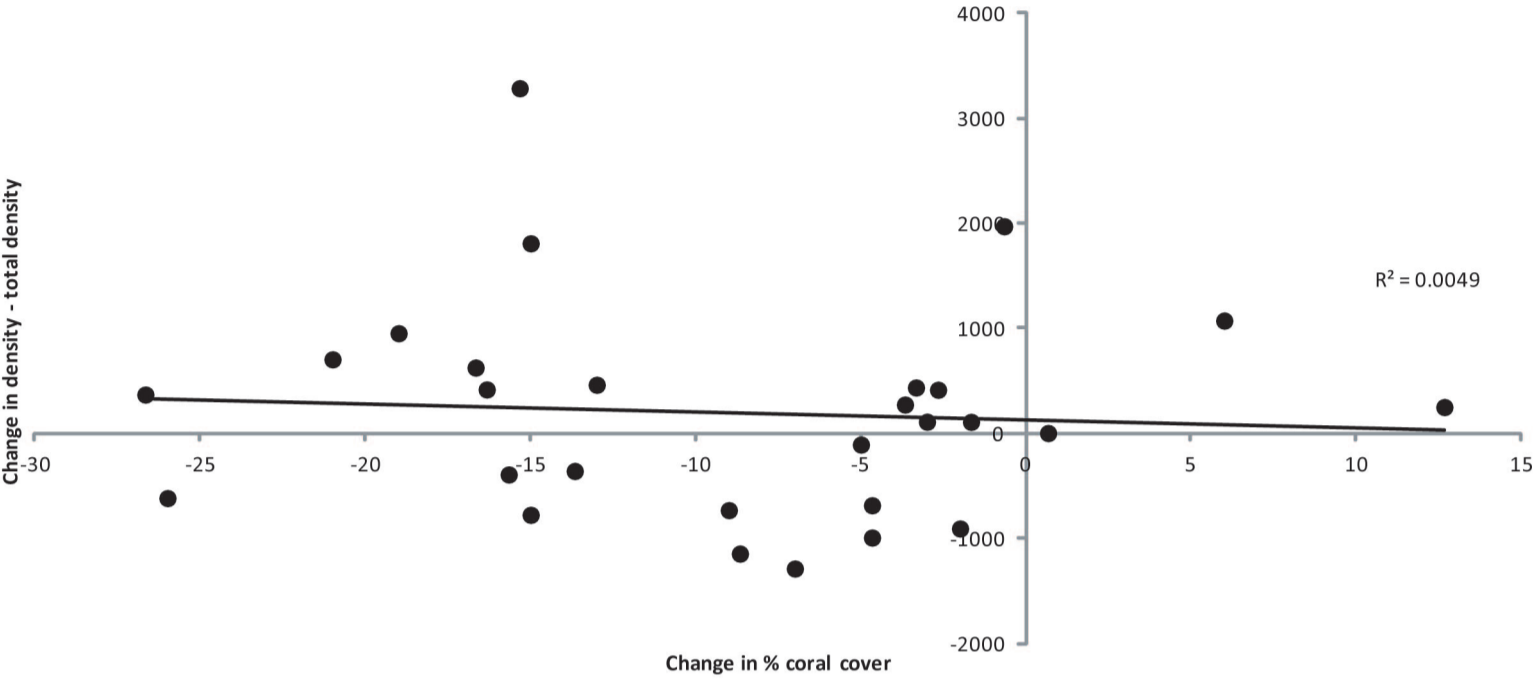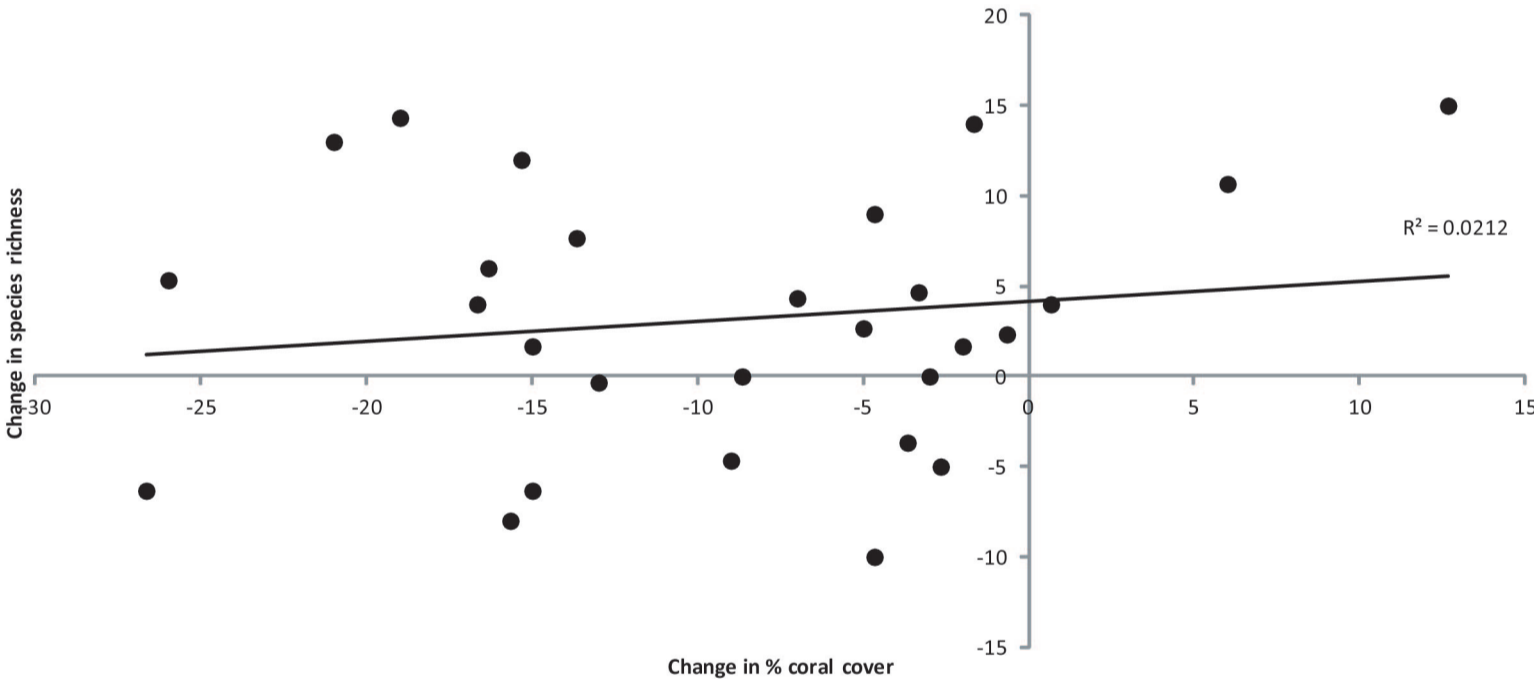

Family level

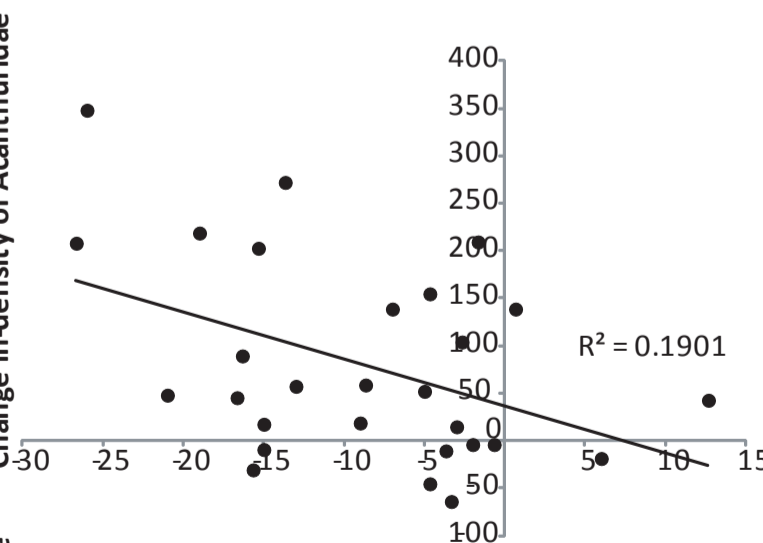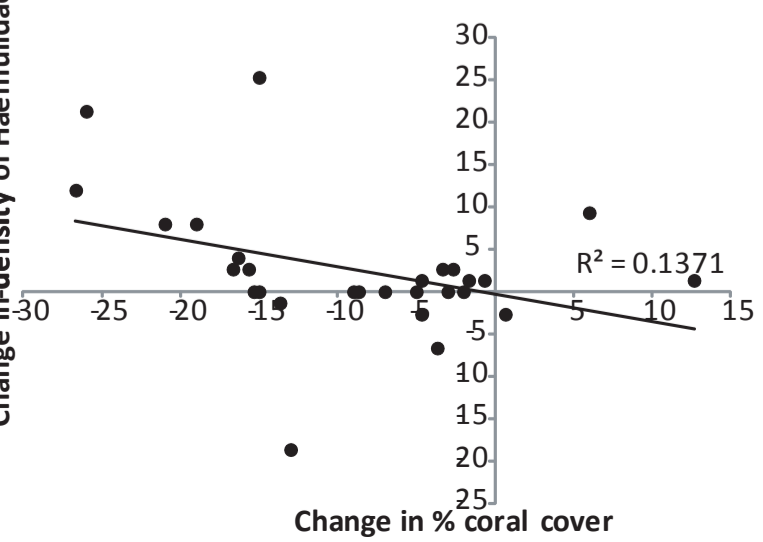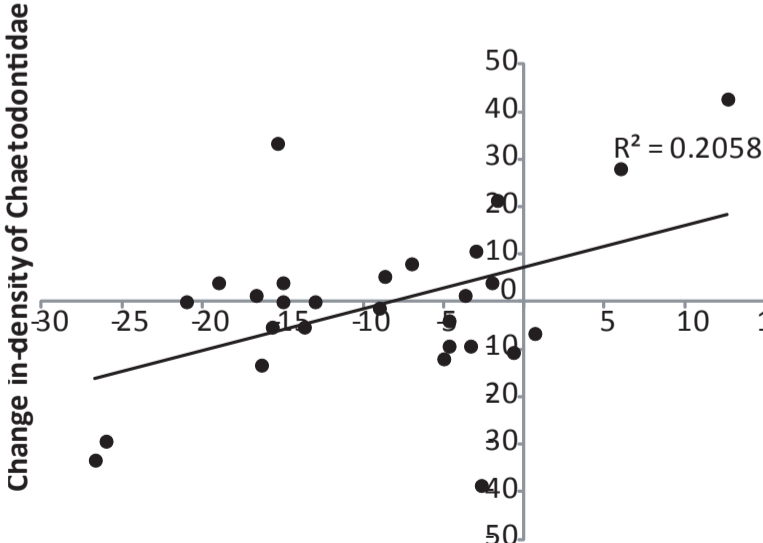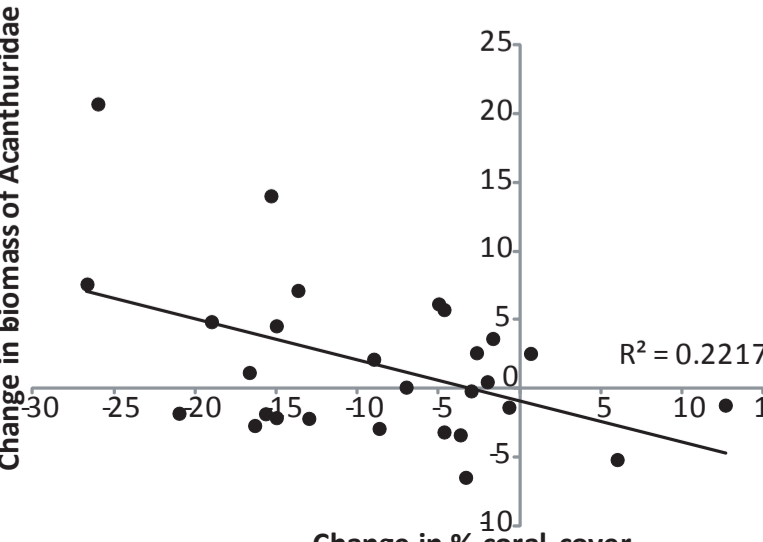

Species level

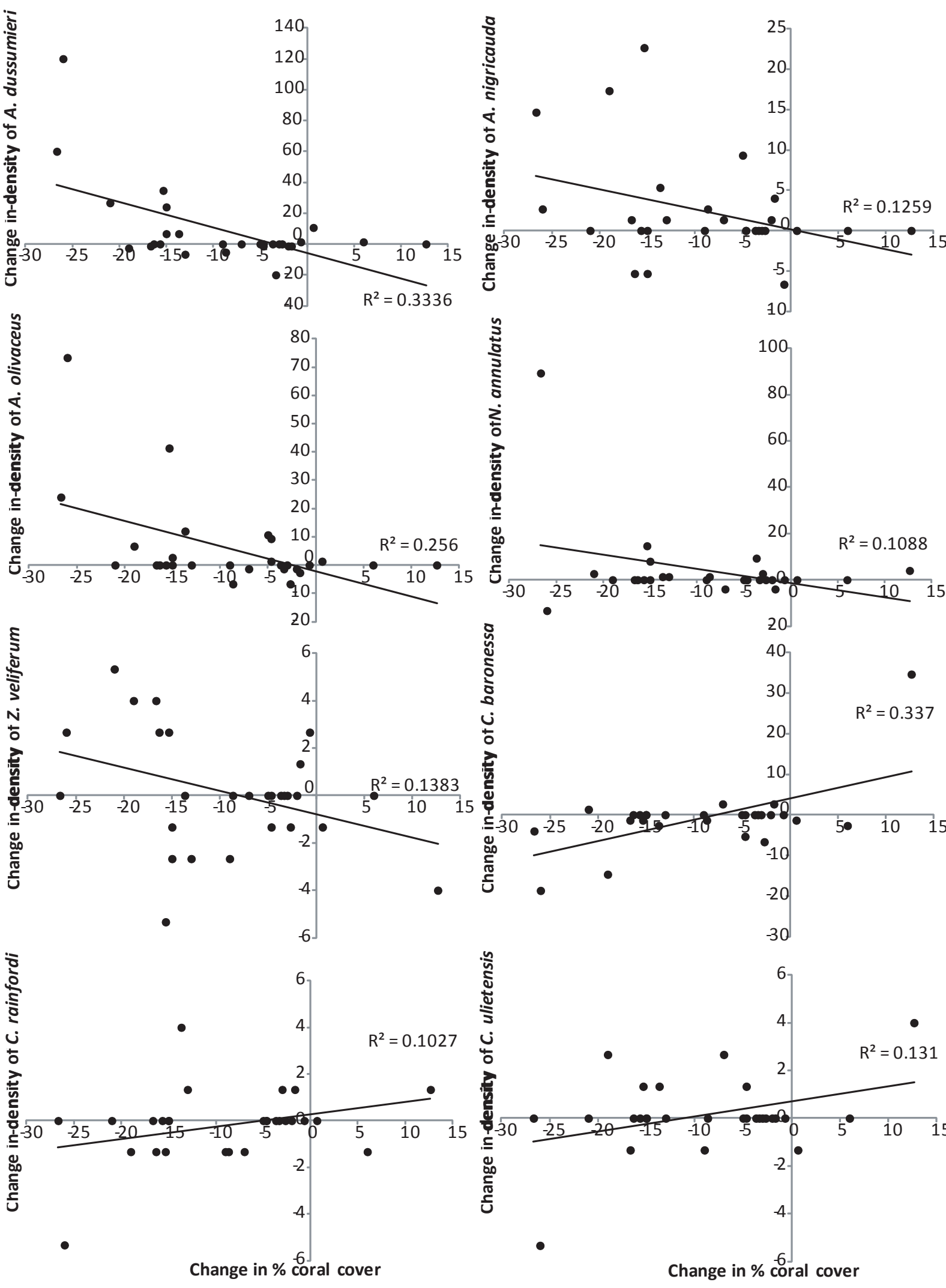

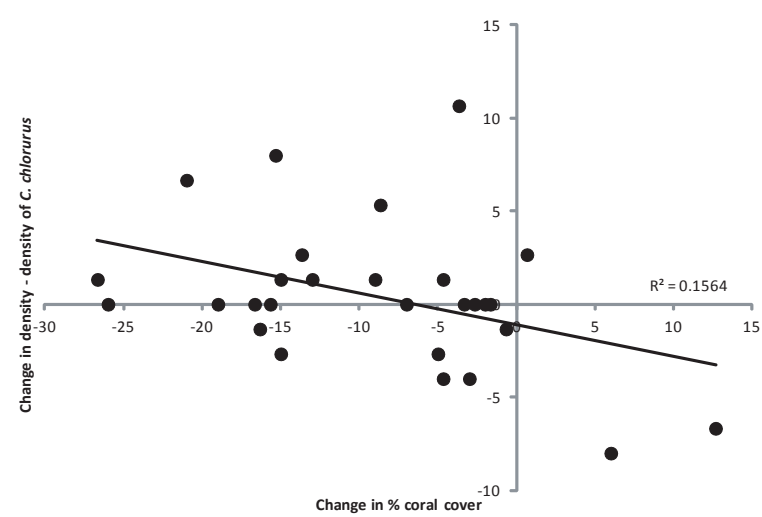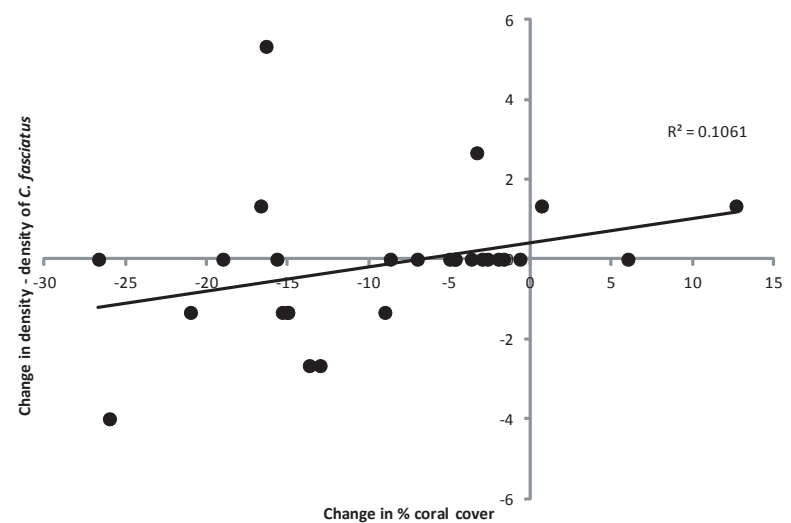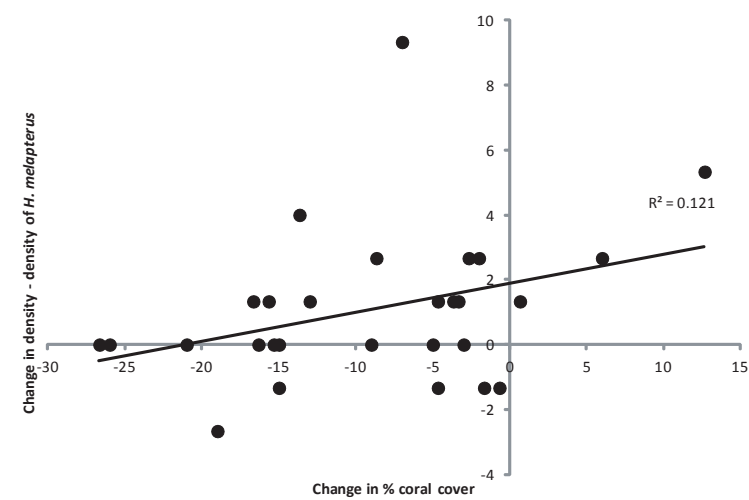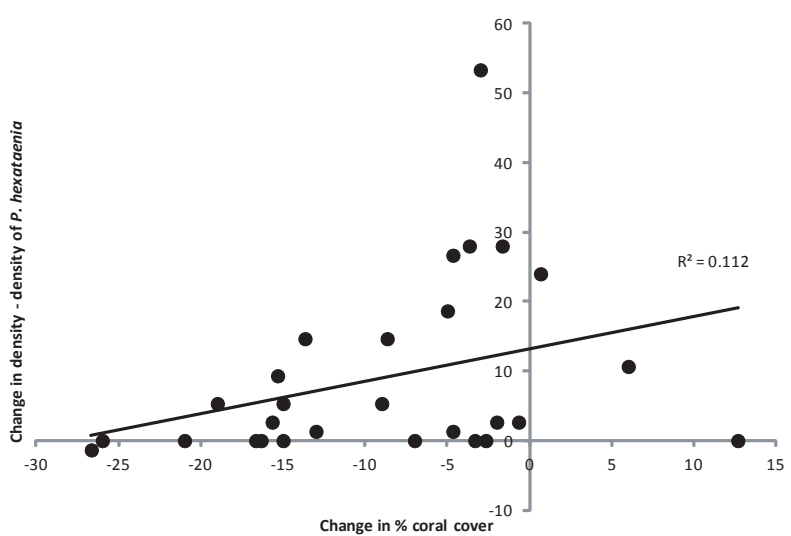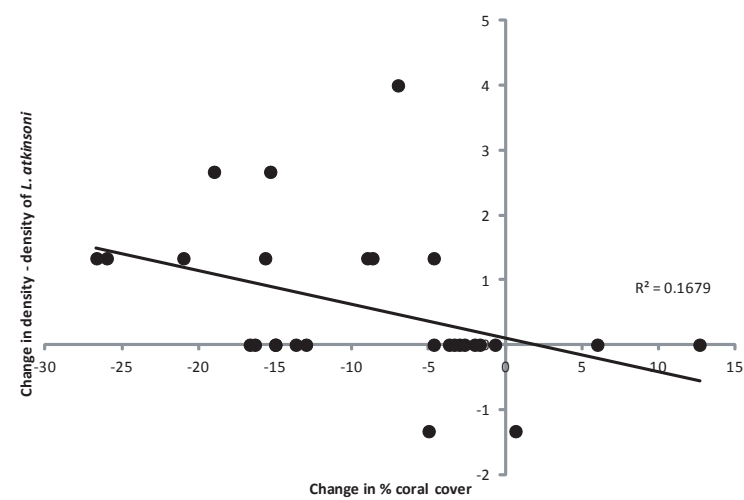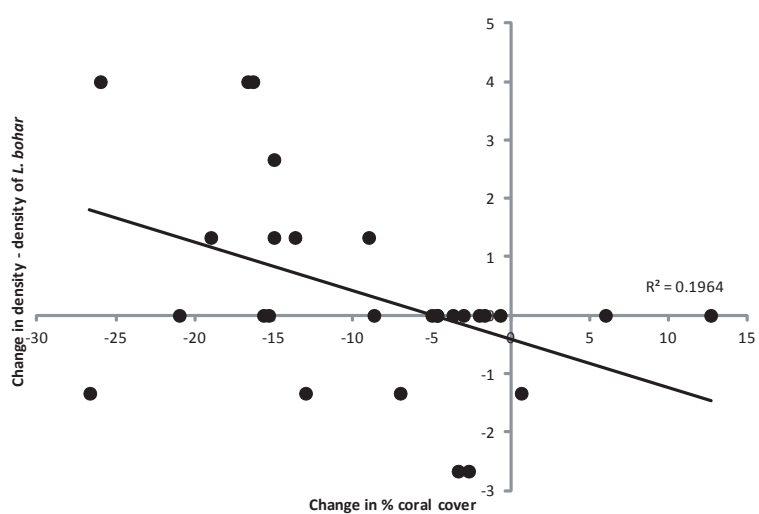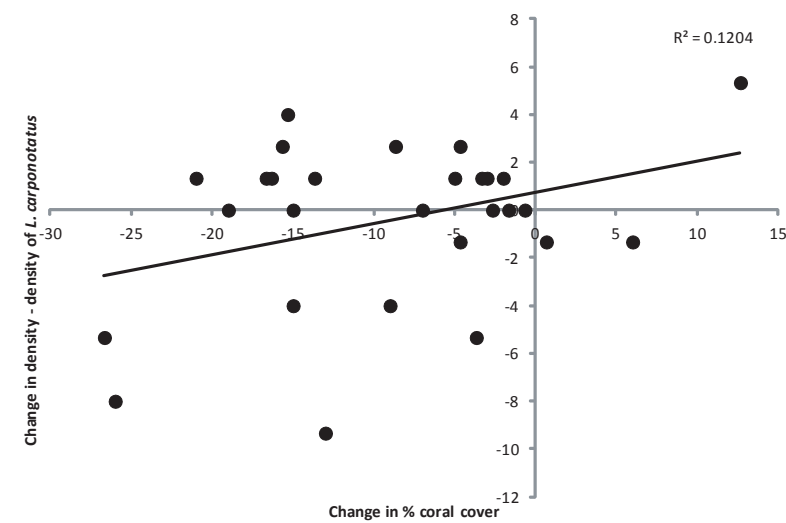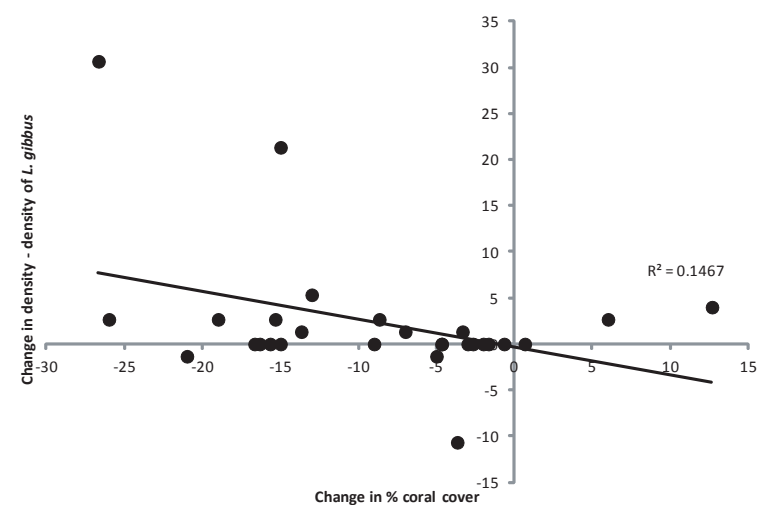

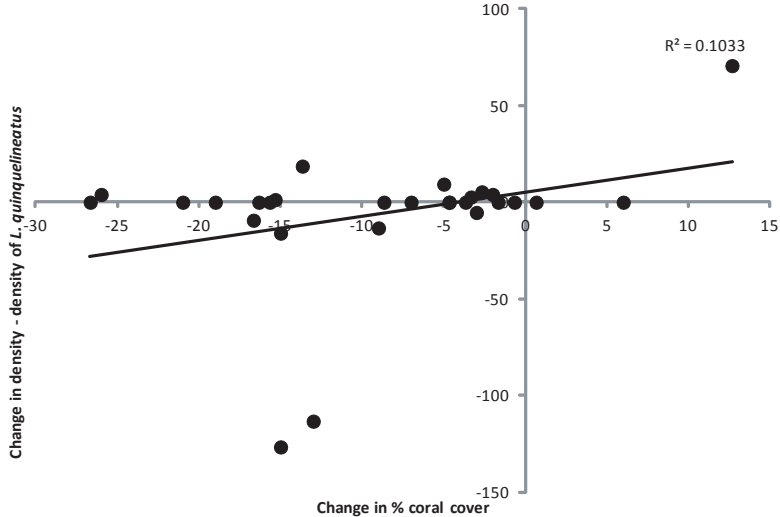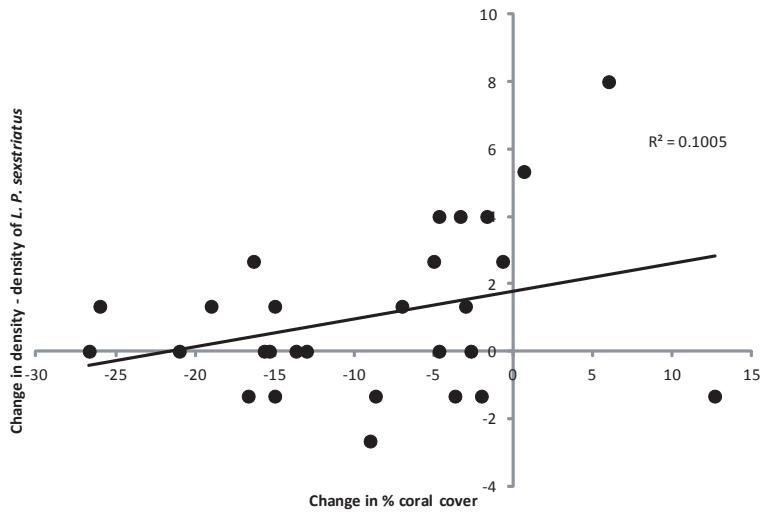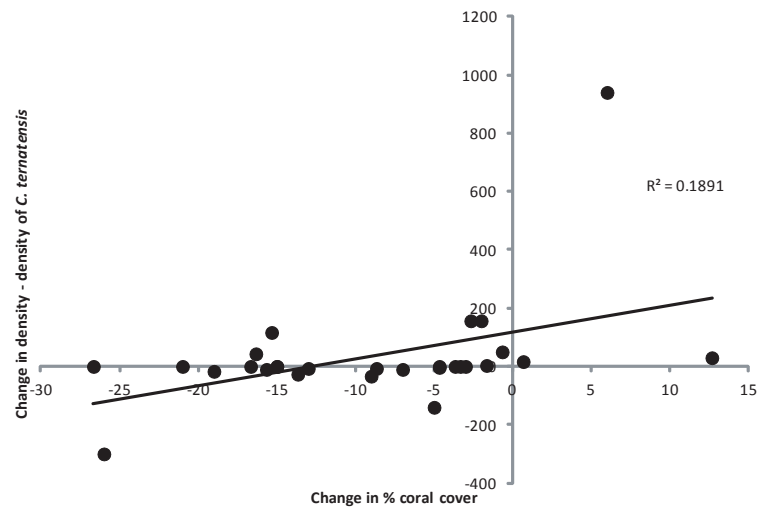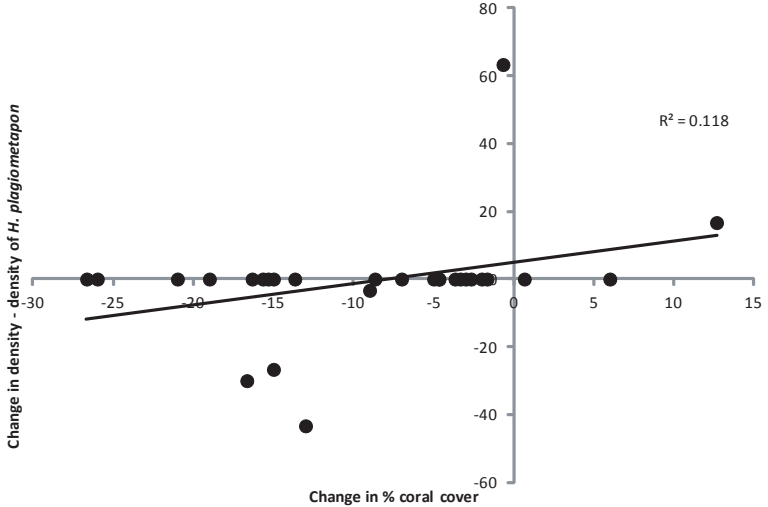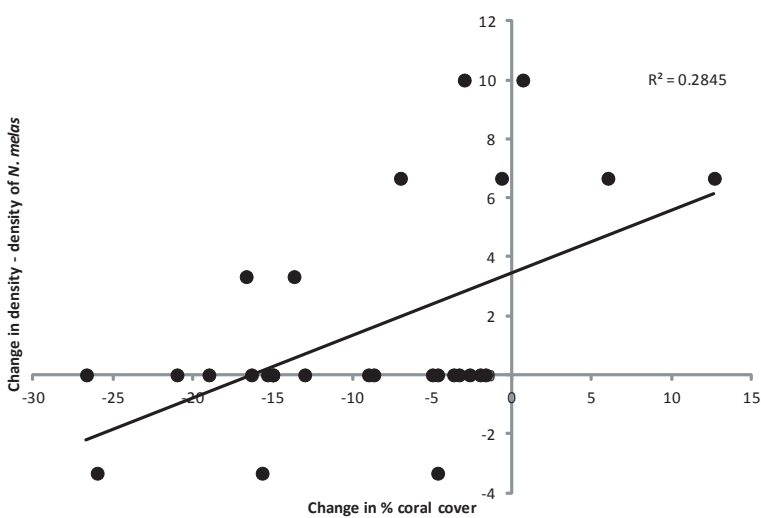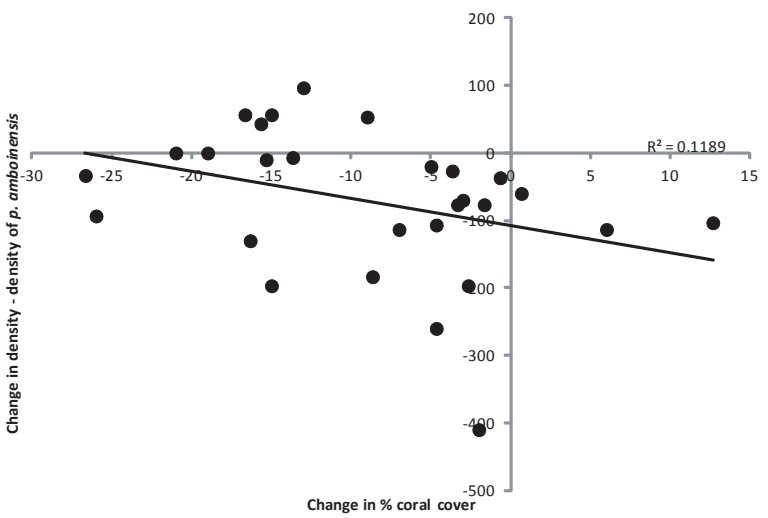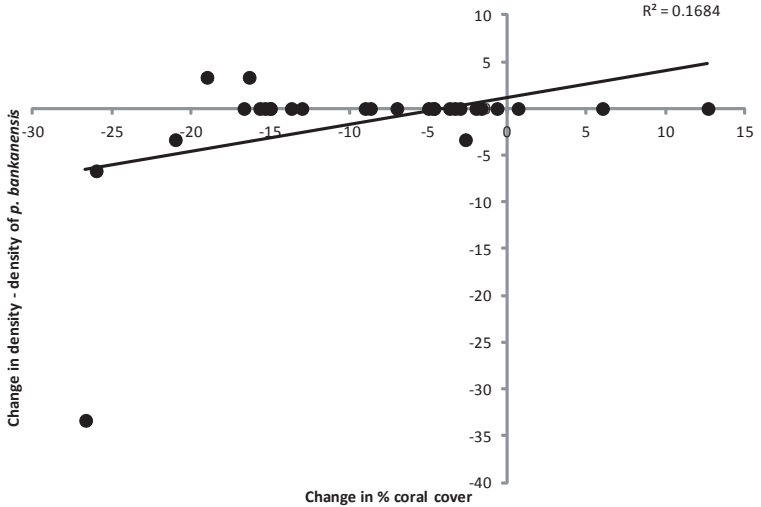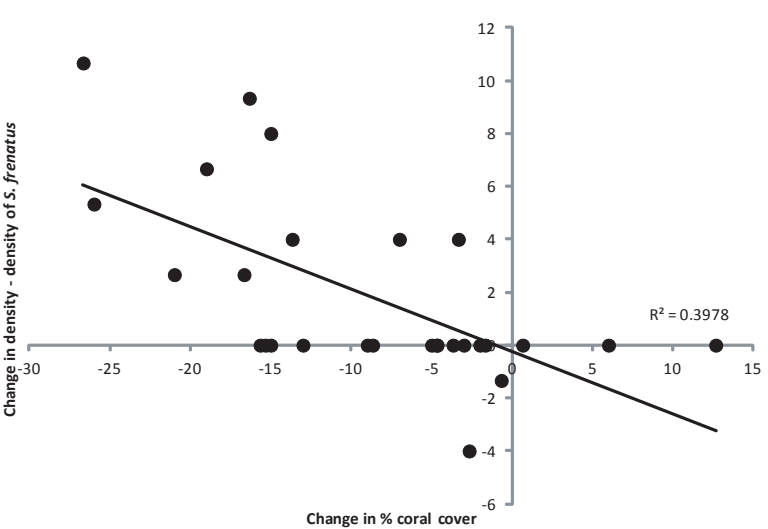

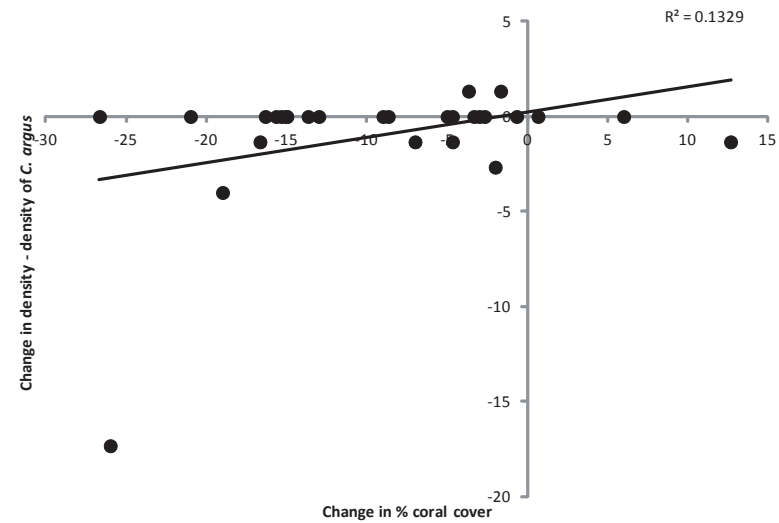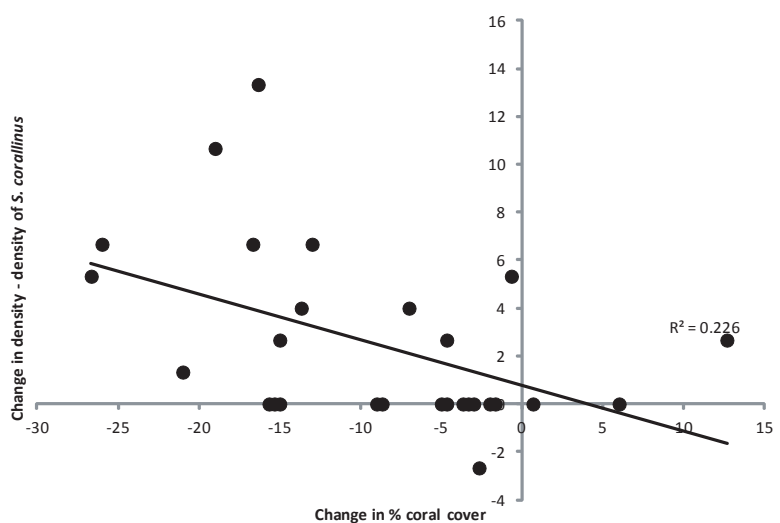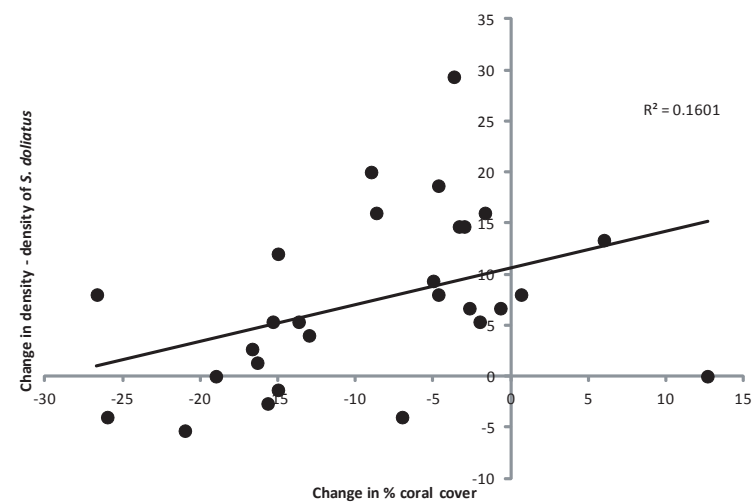

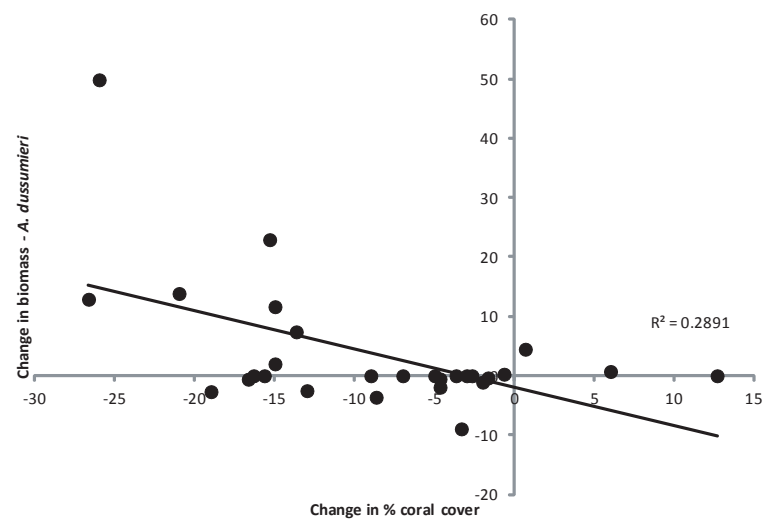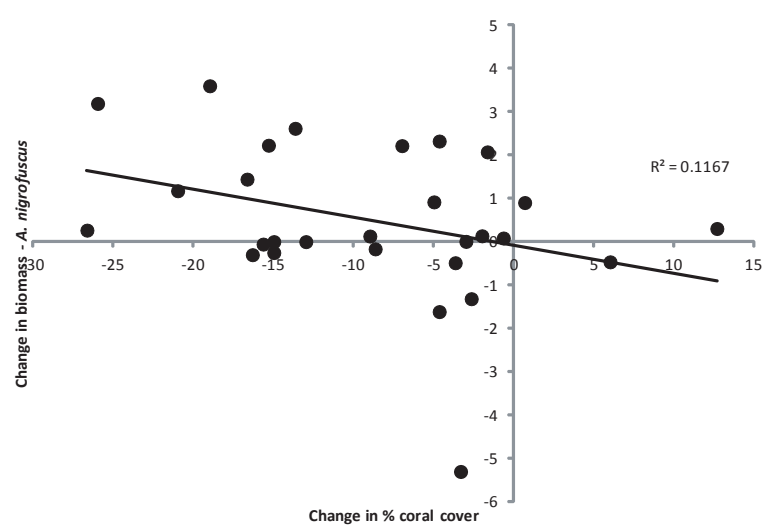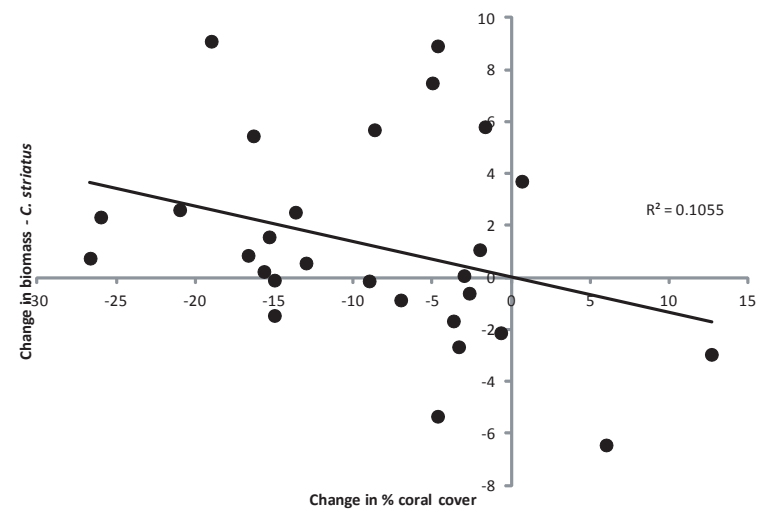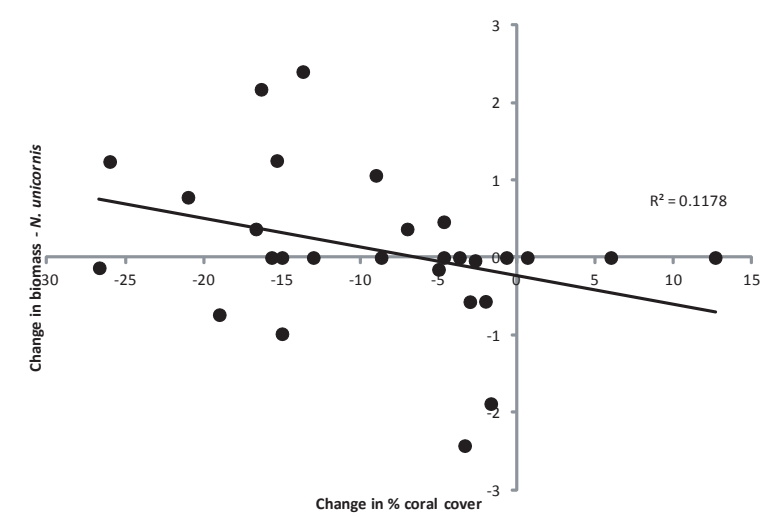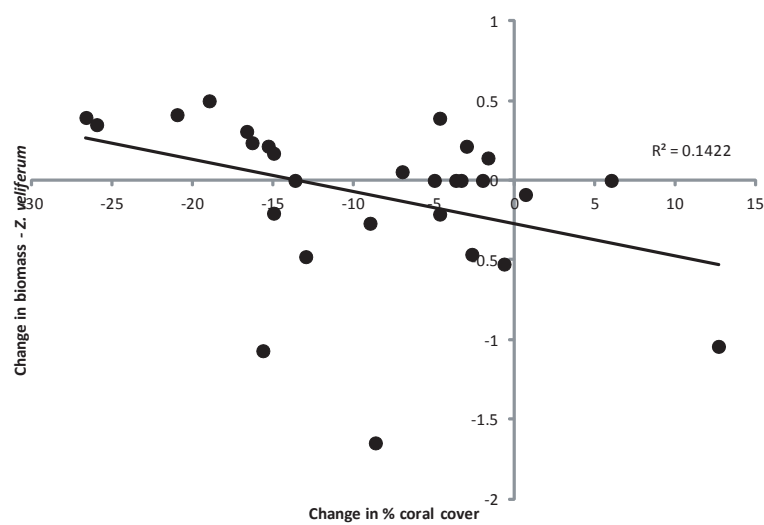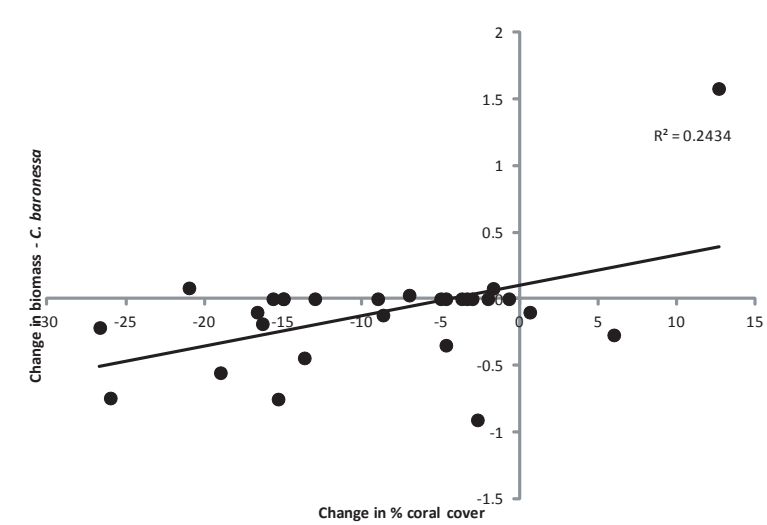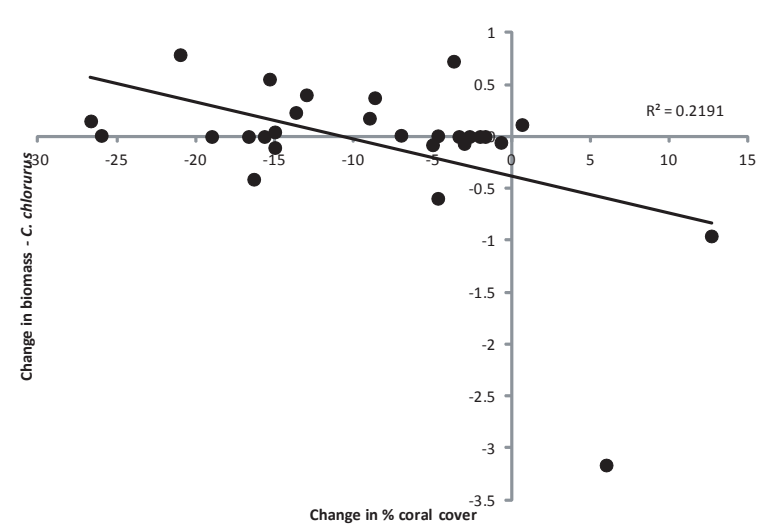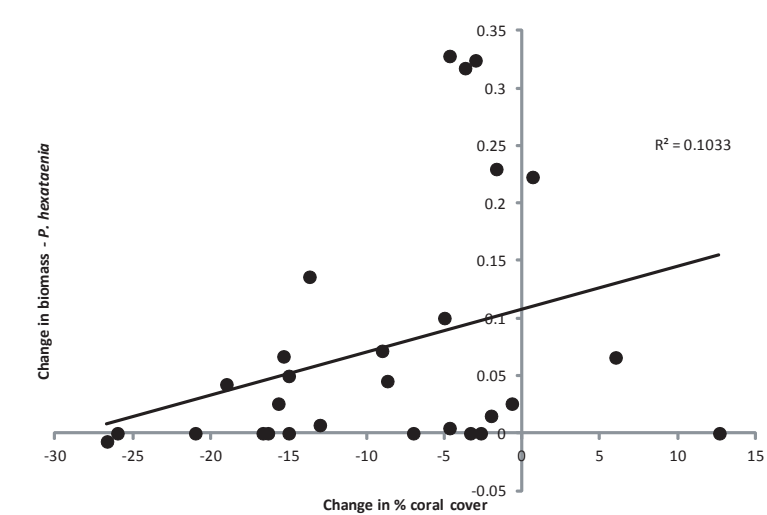

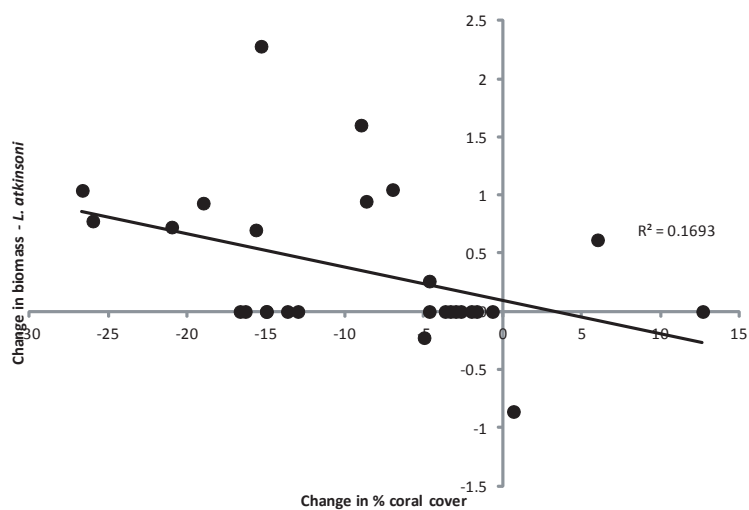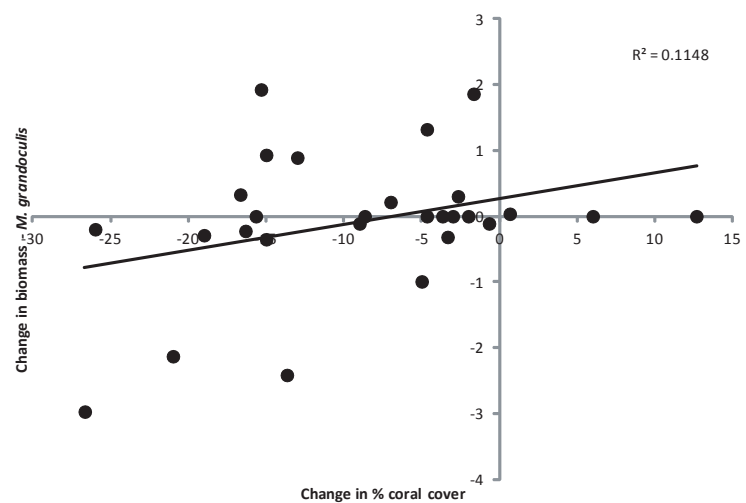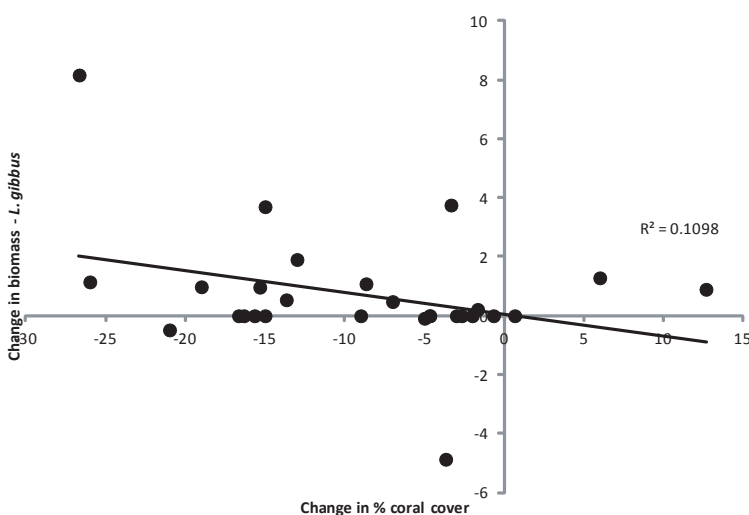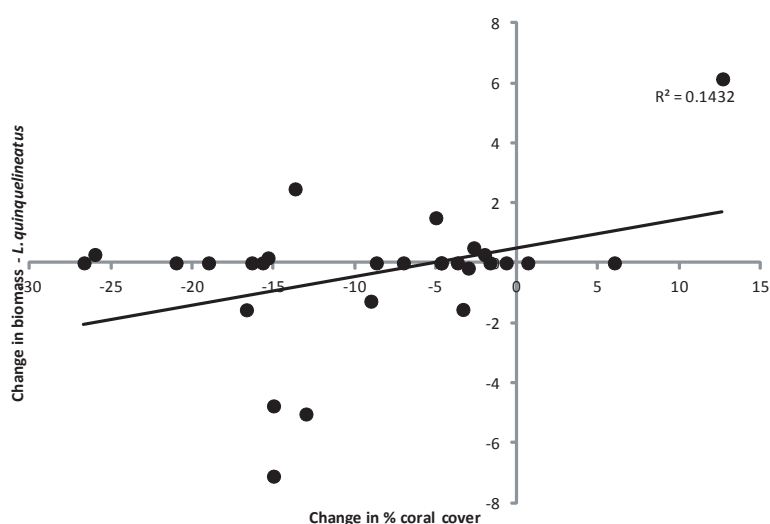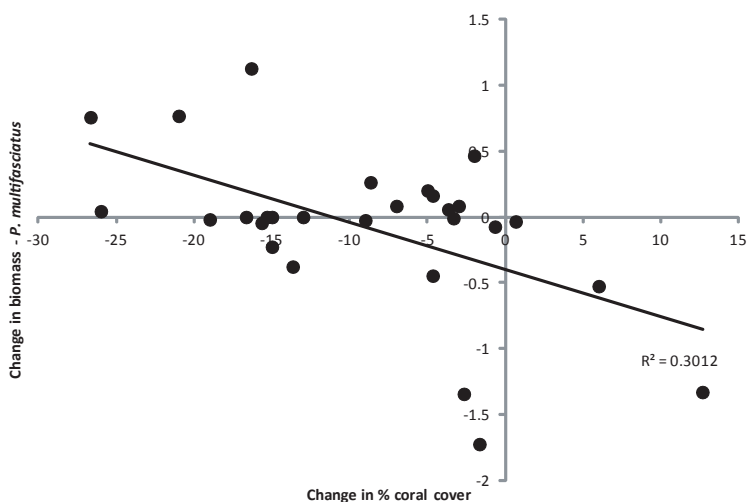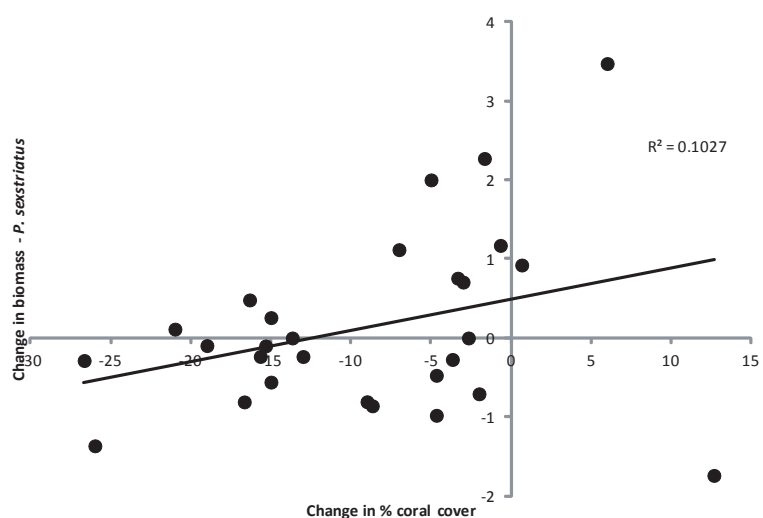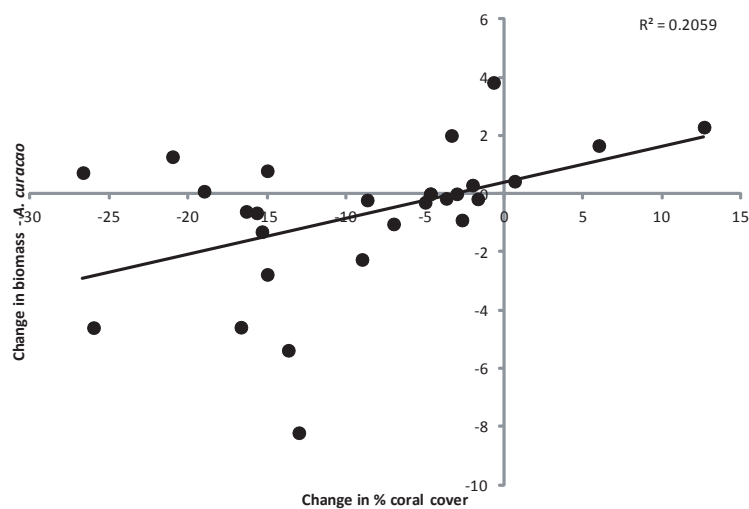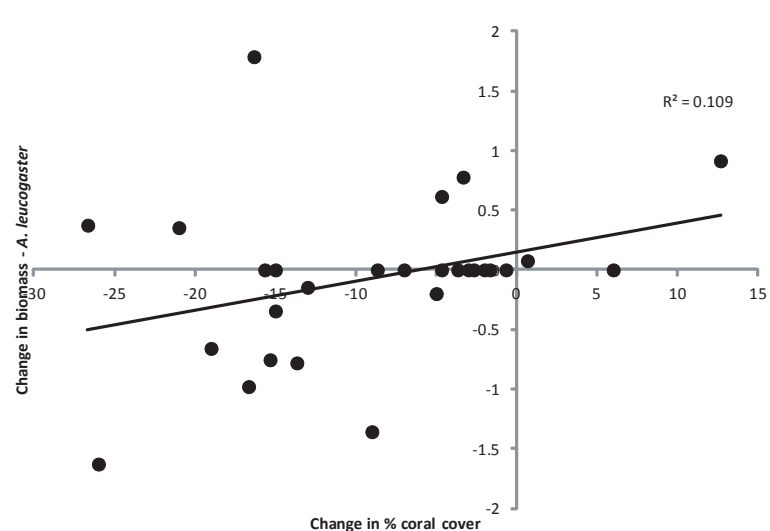

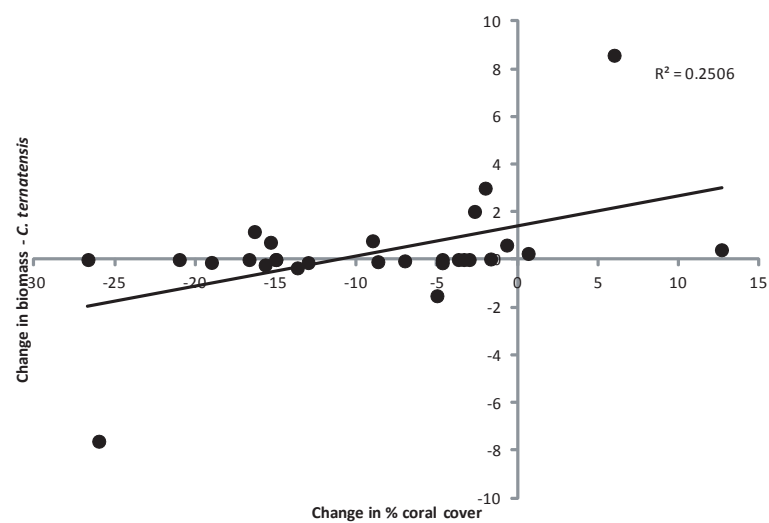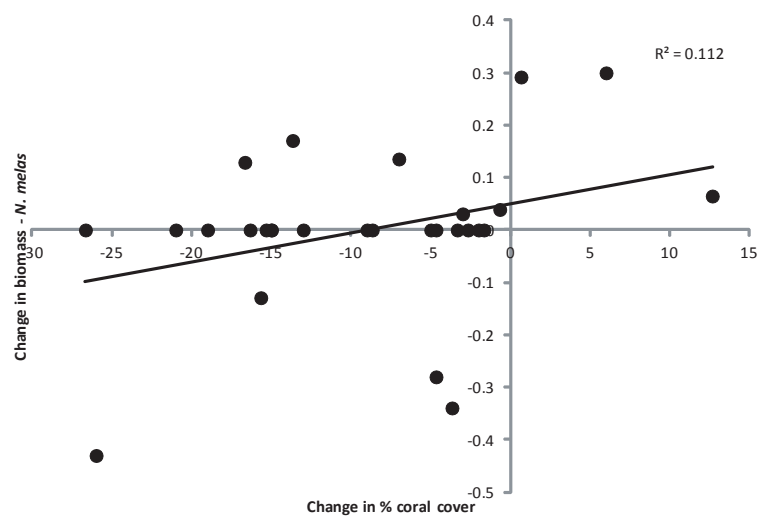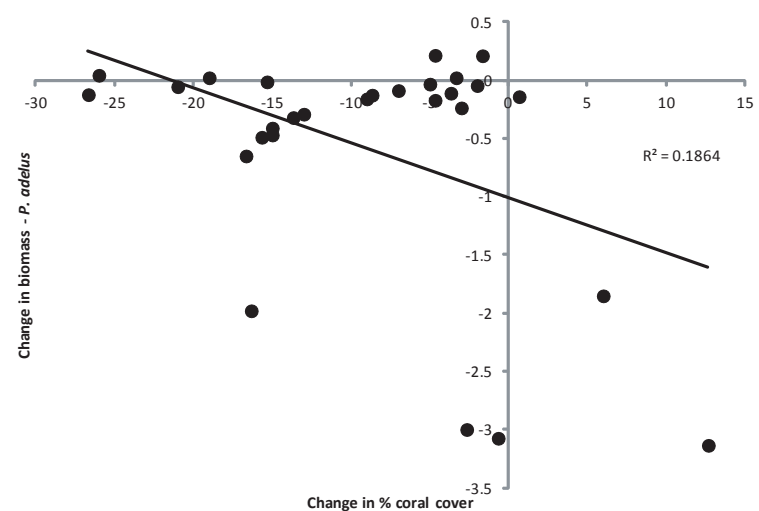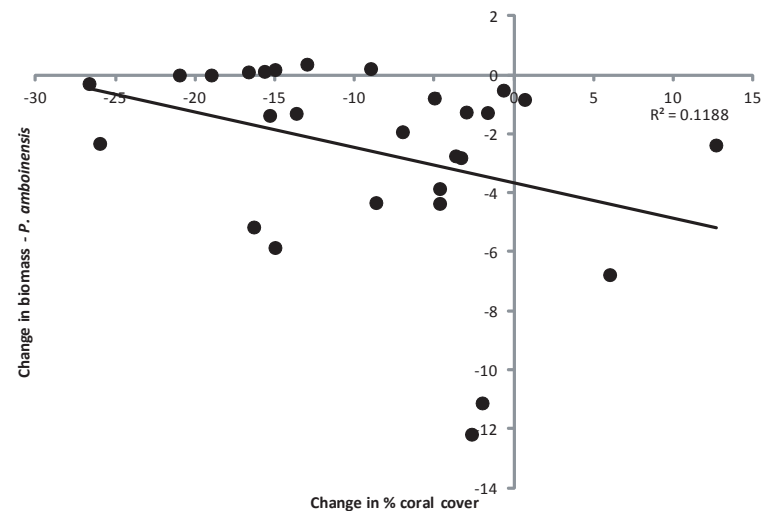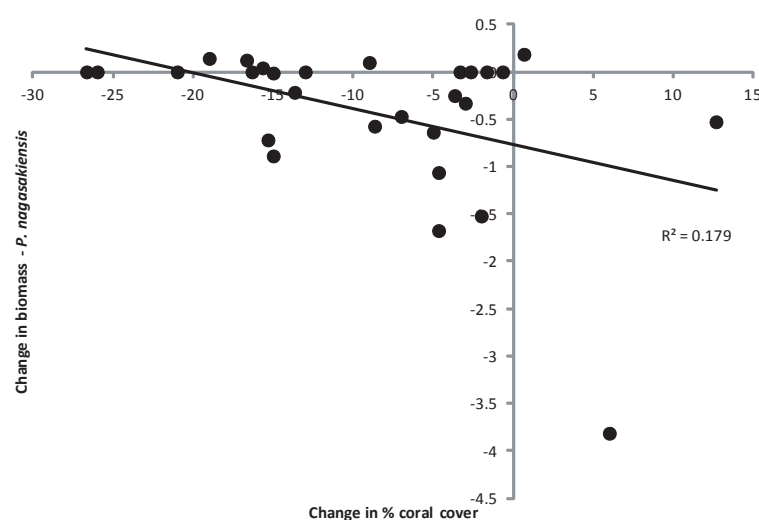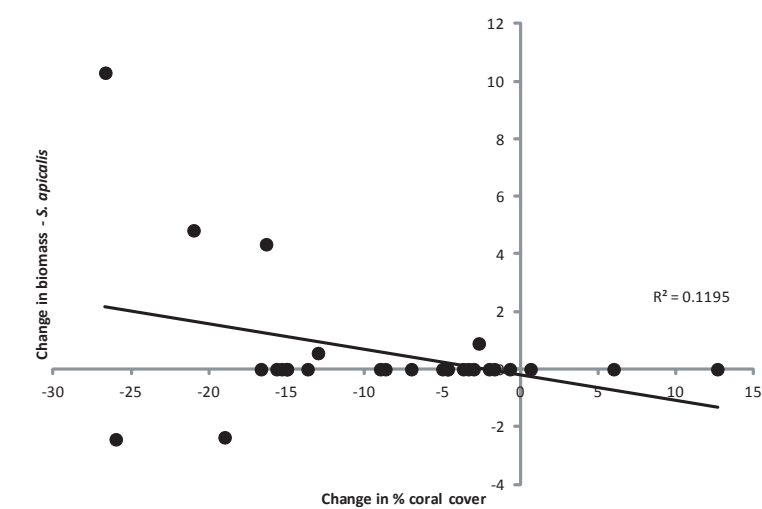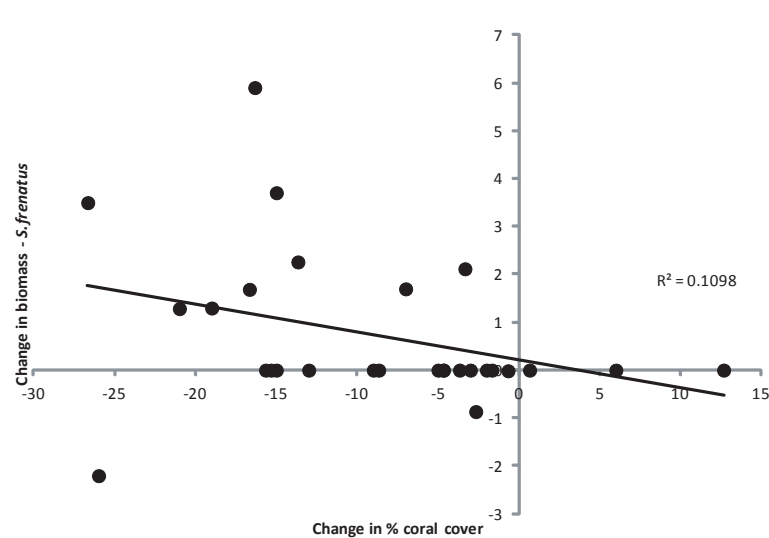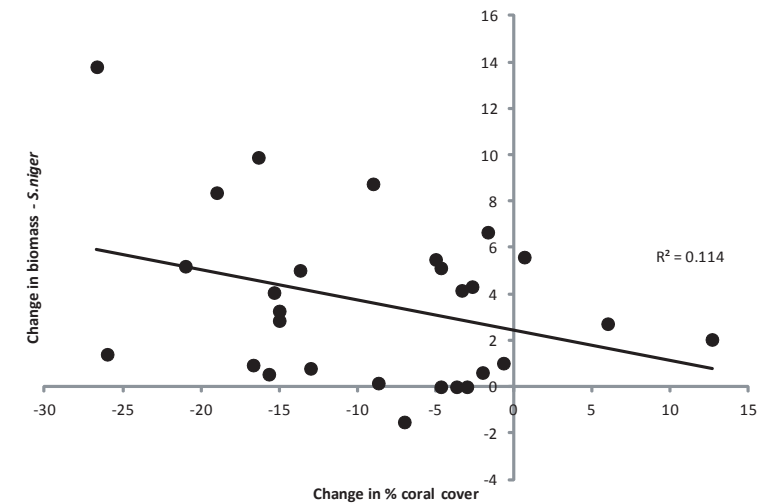

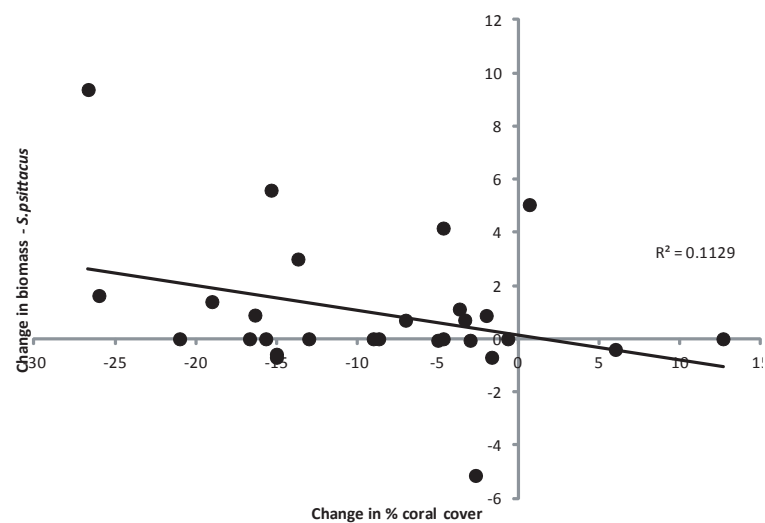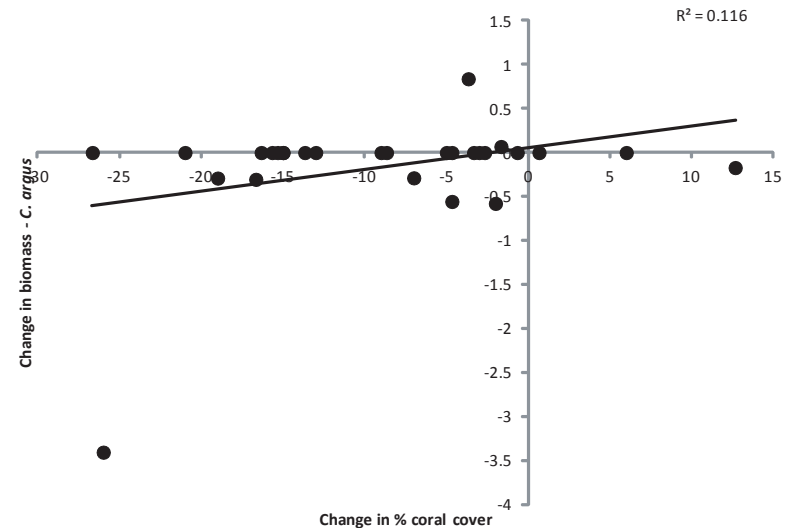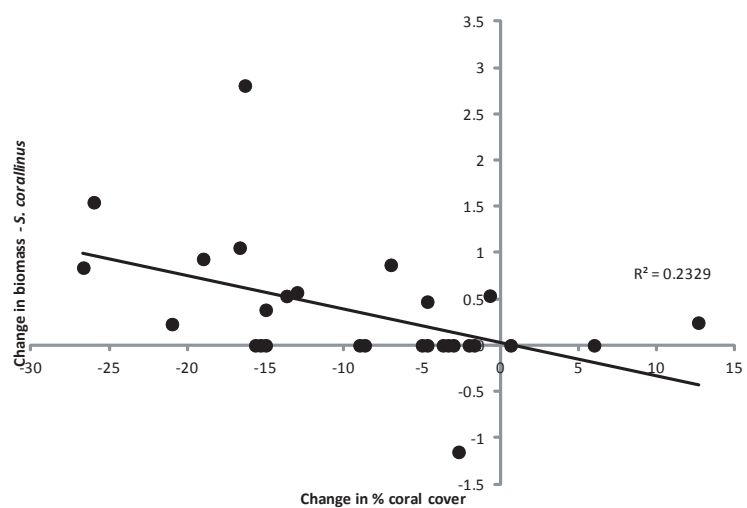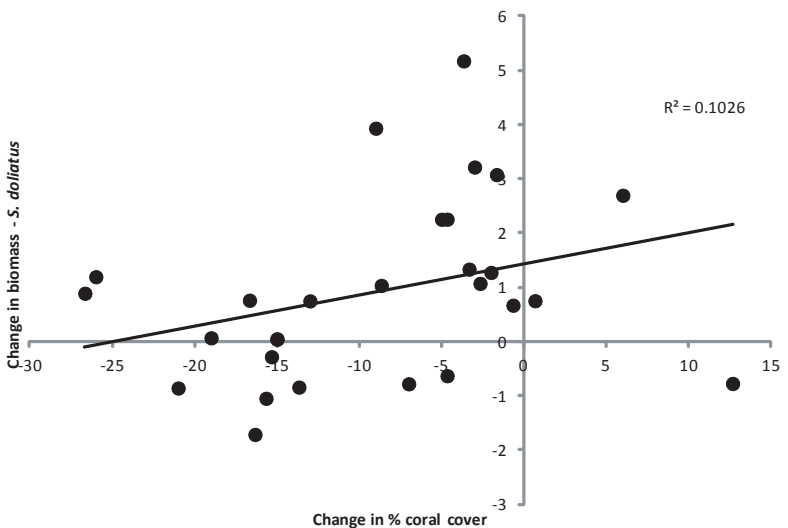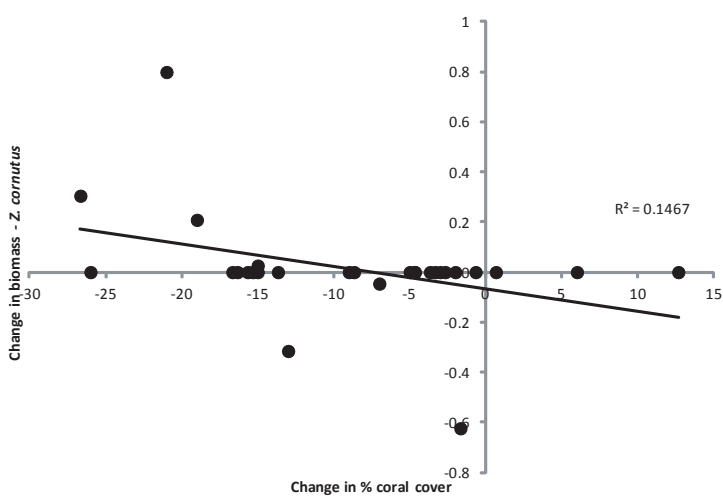

Supplement: S2 Fig — Regressions of coarse metrics, family-level and species-level density and biomass on coral cover. Regressions were performed on the site-depth averages of changes in density and biomass. All summary metrics are shown. For family and species level regressions, only taxa with an R2 of at least 0.1 are given. (PDF) [file pone.0156232.s002.pdf]
