# Supplementary material for: Post-Disturbance Stability of Fish Assemblages Measured at Coarse Taxonomic Resolution Masks Change at Finer Scales
Source: PLoS One. 2016 Jun 10;11(6):e0156232. doi: 10.1371/journal.pone.0156232 (PMC4902313; doi:10.1371/journal.pone.0156232)
Supplement: S3 Fig — Long-term trends in % coral cover, total fish abundance and fish species richness on exposed sites of Lizard Island (red line), MacGillivray Reef (green line) and North Direction Reef (blue line), showing relative stability in coral and fish communities in the 10 years before Cyclone Ita. (PDF) [file pone.0156232.s003.pdf]

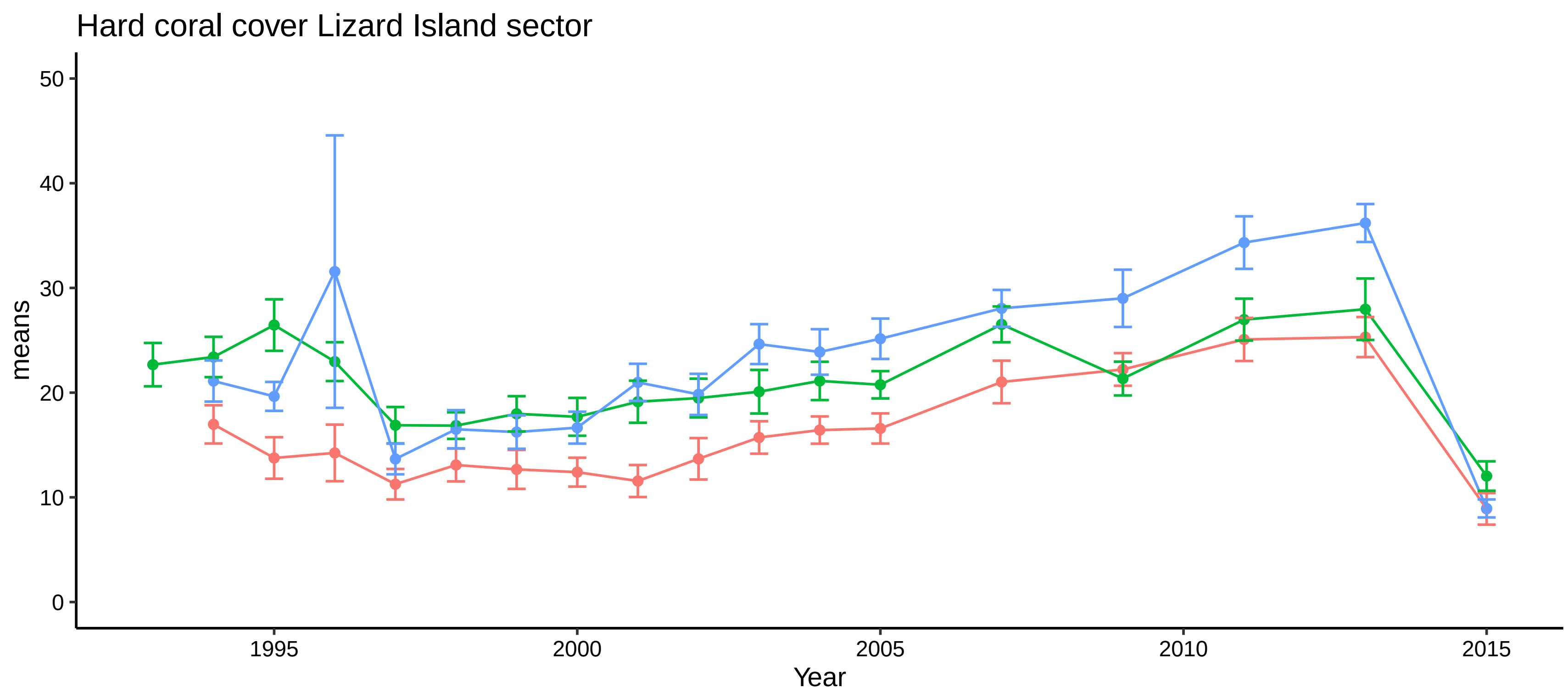

Hard coral cover Lizard Island sector
means
Year

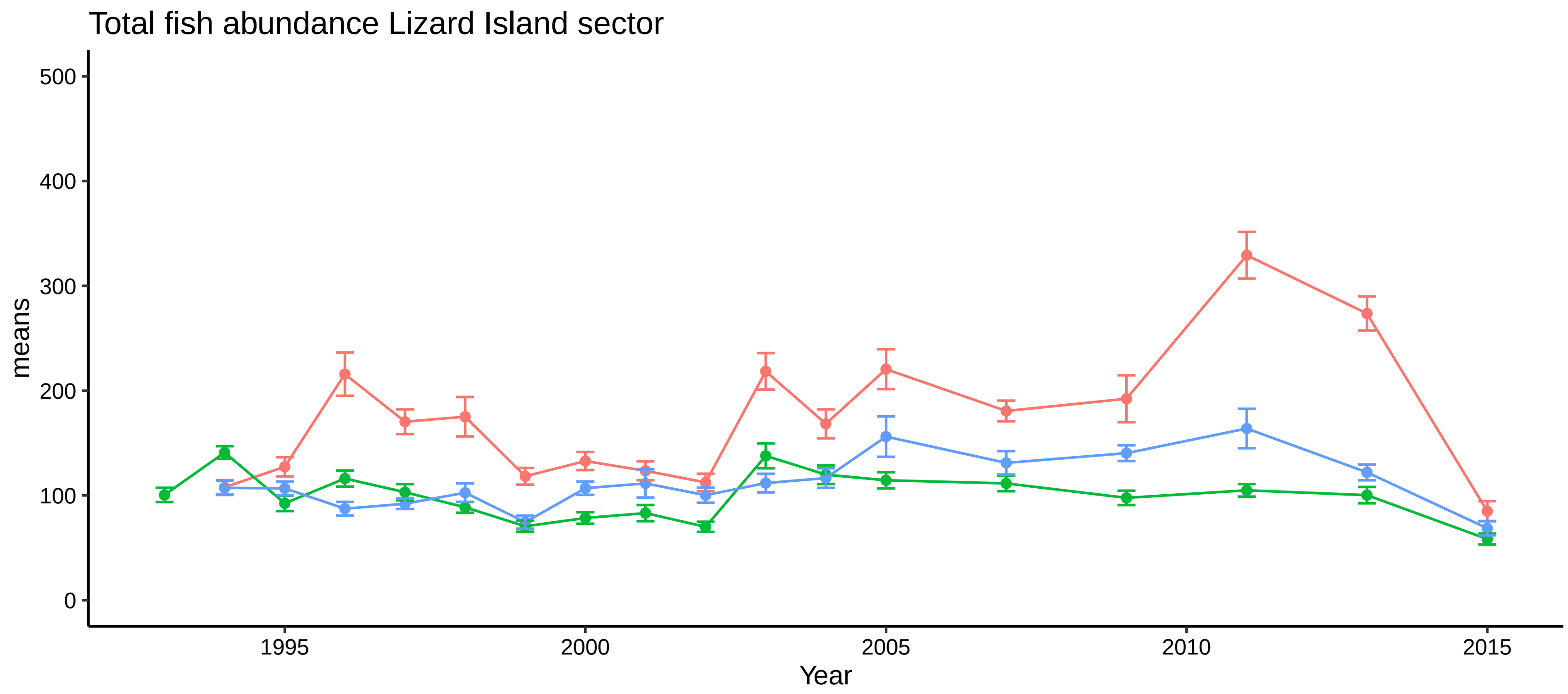

Total fish abundance Lizard Island sector
means
Year

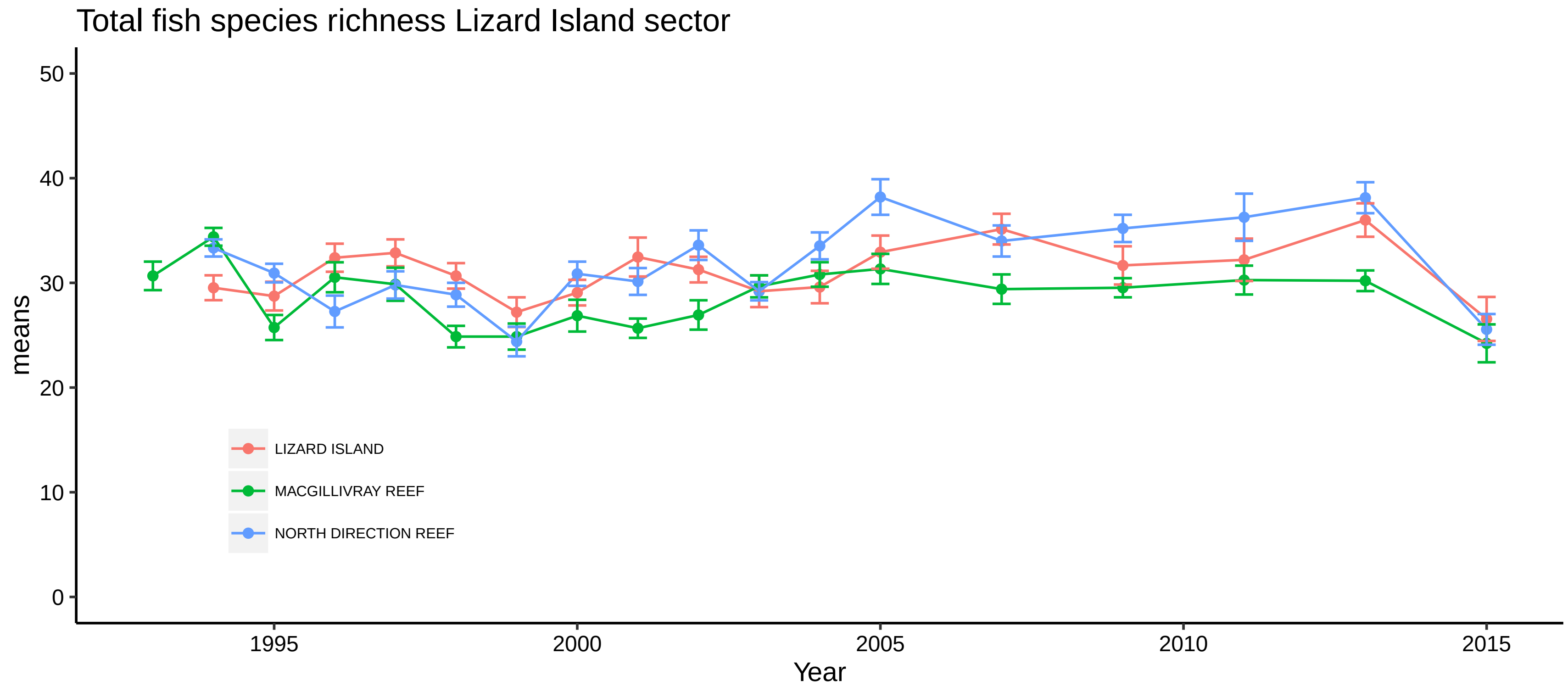

Total fish species richness Lizard Island sector
means
Year
LIZARD ISLAND
MACGILLIVRAY REEF
NORTH DIRECTION REEF
